# Engineering antisense oligonucleotides for targeted mRNA degradation through lysosomal trafficking†

**DOI:** 10.1039/d5sc03751d

**Published:** 2025-06-09

**Authors:** Disha Kashyap, Thomas A. Milne, Michael J. Booth

**Affiliations:** a Department of Chemistry, University of Oxford Mansfield Road Oxford OX1 3TA UK m.j.booth@ucl.ac.uk; b MRC Molecular Haematology Unit, MRC Weatherall Institute of Molecular Medicine, Radcliffe Department of Medicine, University of Oxford Oxford OX3 9DS UK thomas.milne@imm.ox.ac.uk; c Department of Chemistry, University College London 20 Gordon Street London WC1H 0AJ UK

## Abstract

Antisense oligonucleotides (ASOs) can modulate gene expression at the mRNA level, providing the ability to tackle conventionally undruggable targets and usher in an era of personalized medicine. A key mode of action for ASOs relies upon RNase H-engagement in the nucleus, however, most mature mRNA is present in the cytoplasm. This disconnect limits the efficacy and biomedical applications of ASOs. In this paper, we have established a new mechanism of action for achieving potent and targeted mRNA knockdown by leveraging a lysosomal degradation pathway. To achieve this, we employ autophagosome-tethering compound (ATTEC) technology that utilises bifunctional small molecules for lysosomal trafficking. In this manner, to induce degradation of target mRNA located in the cytoplasm, we conjugated an ATTEC warhead, ispinesib, to RNase H-inactive ASOs. These fully 2′-*O*-methylated RNase H-inactive ASOs have higher chemical stability and tighter mRNA binding than conventional ‘gapmer’ sequences, but cannot be recognised by RNase H. Using our lysosomal trafficking antisense oligonucleotide (LyTON) technology, we show significant lysosome-dependent knockdown of multiple molecular targets in various cell lines, *via* transfection and gymnotic uptake. The LyTON modification is also able to boost the knockdown efficacy of RNase H-active ‘gapmer’ ASOs. Engineered to degrade mRNA independent of RNase H recognition, LyTONs will enable gene silencing using oligonucleotide chemistries with higher chemical stability, tighter mRNA binding affinity, and improved cell delivery profiles. This will enable us to target a wider range of disease-relevant mRNA, potentially leading to the development of new therapies.

## Abstract 2

Lysosomal-trafficking antisense oligonucleotides (LyTONs) allow for the lysosomal degradation of target mRNA-enabling potent gene silencing in the cytoplasm, *via* an RNase H-independent mechanism.

## Graphical abstract

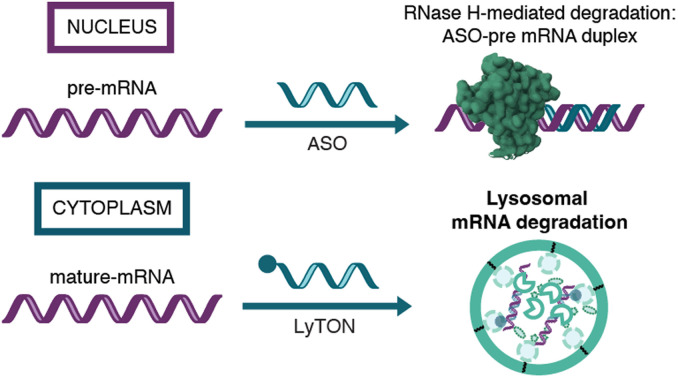

## Introduction

Nucleic acid drugs are promising therapeutic modalities due to their unique ability to target genetic pathways with exquisite specificity.^1^ Antisense oligonucleotides (ASOs) lead the charge with over 10 drugs approved in the past two decades.^2^ ASOs are short synthetic strands of DNA designed to modulate gene expression by binding to target mRNA through base-pair complementarity. Approved ASO drugs operate through two main mechanisms: the ASO-RNA duplex can either modulate pre-mRNA splicing or trigger RNase H-mediated degradation.^3^ However, despite their success, approved ASOs target a narrow spectrum of diseases and their key mechanisms of action are predominantly localised within the nucleus.

Pre-mRNA splicing requires a specialized ASO mechanism active in the nucleus.^4^ This process requires ASOs to bind pre-mRNA in the nucleus and ultimately influence splicing into mature mRNA. This mechanism is a powerful strategy for treating genetic diseases caused by splicing defects. In contrast, general mRNA knockdown mechanisms are constrained by limited RNase H recruitment^5^ – RNase H is enriched in the nucleus, whereas mature mRNA is located in the cytoplasm. Thus, RNase H recruitment is unreliable as its expression levels vary not only with cellular localisation but also with cell and tissue type.^6^ One approach to improve target engagement—and thus the efficacy—of ASO-based gene knockdown therapies is to address this localization mismatch between the effector pathway and its molecular target. Conjugation with a nuclear importer has been shown to increase the activity of splice-switching oligonucleotides and RNase H-active ASOs.^7^ However, this mechanism may not be desirable for different mRNA targets. RNase L-mediated cytoplasmic degradation of viral RNA has been achieved through the covalent modification of ASOs with endogenous RNase L ligands.^8^ However, fine control over RNase L activity is key; uncontrolled activation of this potent cytoplasmic anti-viral effector pathway can lead to global translational shutdown,^9^ amplification of cellular stress responses, and cell death.^10^ This demonstrates the need to develop new methods to harness a selective cytoplasmic ASO pathway for targeted degradation of mRNA.

Lysosomes serve as the primary degradation compartment within cells,^11^ capable of breaking down a wide range of biomolecules.^12–14^ This functionality has been harnessed for protein degradation using antibodies, such as LYTACs (lysosome-targeting chimeras),^15,16^ bispecific aptamer chimeras,^17^ and small molecules with approaches such as MrTACs (methylarginine targeting chimeras).^18^ These methods all employ ligands or chemical tags to direct proteins to the lysosome for degradation. Inspired by these strategies, we investigated whether lysosomal trafficking could be exploited for targeted mRNA degradation. For this purpose, we utilized ispinesib, a small molecule originally developed to inhibit the mitotic kinesin Eg5 protein as an anti-cancer drug.^19,20^ Ispinesib has also been employed as a warhead in bi-functional molecules designed to degrade target proteins *via* lysosomal trafficking.^21^ The proposed mechanism for these bi-functional molecules involves LC3-binding within the autophagosome, which subsequently fuses with the lysosome to mediate protein degradation. This activity was lost in the presence of inhibitors targeting various steps of autophagy or lysosomal trafficking. Bi-functionals with this mode of action are named as autophagosome-tethering compounds (ATTECs).^22^

**Fig. 1:**
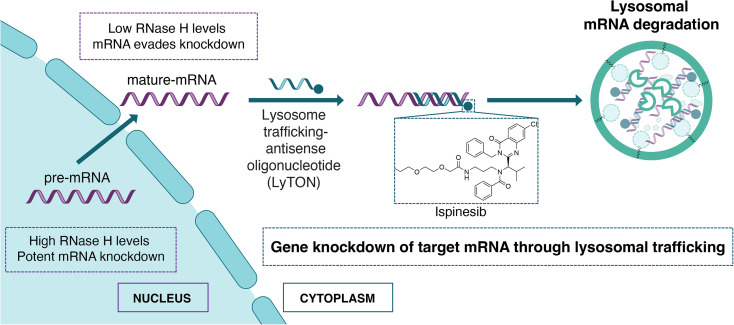
Ispinesib-ASO conjugates act as lysosomal trafficking antisense oligonucleotides (LyTONs), enabling targeted mRNA degradation in the lysosome.

By conjugating the small molecule ispinesib to ASOs, we have developed lysosomal trafficking antisense oligonucleotides (LyTONs) that can selectively knockdown mRNA through transport to the lysosome (Fig. 1). Inhibition of lysosomal activity, *via* bafilomycin or chloroquine, resulted in the loss of LyTON activity, demonstrating that LyTON-mediated transport of mRNA to the lysosome was responsible for knockdown. To illustrate the promise of this technology, LyTONs were developed to target Menin (MEN1), a promising clinical target involved in transcriptional regulation over cellular differentiation and proliferation in leukemias.^23^ The MEN1 LyTONs resulted in a dramatic protein-level knockdown following treatment.

By leveraging lysosomal trafficking, this approach provides a powerful mechanism for degrading mRNA without interfering with native cellular processes or triggering widespread cellular damage. The ability to degrade disease-relevant mRNAs in the lysosome will improve target engagement, as nuclear localisation and subsequent RNase H recruitment will no longer be a limiting factor. Currently, all nucleic acid-mediated gene knockdown technologies rely upon DNA (ASOs) or RNA (small interfering RNA, siRNA) backbones for enzymatic recruitment and activity.^24^ In contrast, our LyTONs can be employed with alternative oligonucleotide chemistries that offer higher potency and more favourable pharmacological profiles. Thus, we believe that LyTONs will allow for the targeting of a broader spectrum of clinically-relevant gene targets, expanding our toolkit for developing innovative nucleic acid therapeutics.

## Results

**Fig. 2:**
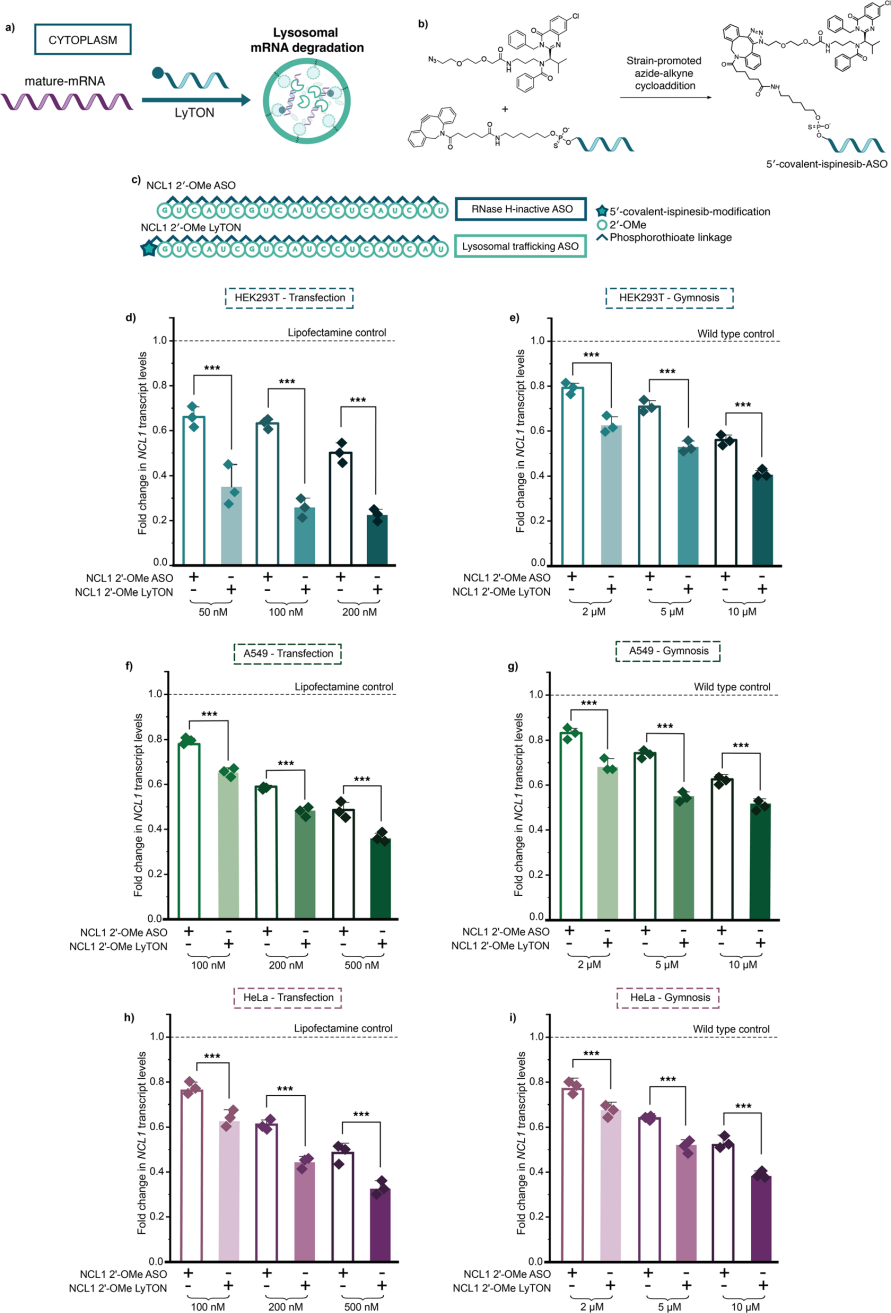
Covalent ispinesib modification of RNase H-inactive 2′-OMe ASO enables knockdown of target mRNA. (a) Proposed mechanism of action for the lysosome trafficking-ispinesib ASO (LyTON) conjugates for mRNA knockdown, in contrast to the established RNase H mechanism for mRNA knockdown of ASOs. (b) Synthesis of covalent ispinesib-ASO conjugates using strain-promoted azide-cyclooctyne cycloaddition. (c) Sequence and chemical modifications of NCL1 ASOs used. (d–i) RT-qPCR data for NCL1 knockdown upon lipofectamine transfection (d, f, and h – for 24 hours) or gymnotic delivery (e, g, and i – for 96 hours) of NCL1 2′-OMe LyTON and unconjugated-NCL1 2′-OMe ASO in HEK293T (d and e), A549 (f and g), or HeLa (h and i) cells at concentrations indicated. Three biological replicates in d–i are shown as diamonds for each condition (each from three technical replicates). The vertical bars represent the mean and the error bars the standard deviation. ** represents *p* < 0.05, *** represents *p* < 0.01, n.s. represents *p* values that are not significant – determined *via* unpaired student's *t*-test.

To test whether we could use the ATTEC-warhead ispinesib for lysosomal trafficking and subsequent lysosomal degradation of target mRNA (Fig. 2a), we decided to covalently attach it to an ASO using strain-promoted azide–alkyne click (SPAAC) chemistry^25^ (Fig. 2b). We opted for the fully 2′-*O*-methyl (2′-OMe) sugar modified-backbone for the test ASO. This chemistry is known to be RNase H-inactive, and would allow us to assess the ispinesib-ASO without interference from RNase H-mediated degradation (Fig. 2c). We chose a previously validated ASO sequence designed to target cytoplasmic NCL1 mRNA.^26^ A 5′-dibenzocyclooctyne (DBCO) NCL1 ASO was synthesised by reacting a 5′-terminally modified-amino 2′-OMe ASO with excess DBCO-*N*-hydroxysuccinimide (NHS) ester. We then used strain-promoted azide–alkyne click (SPAAC) chemistry^25^ to couple commercially available ispinesib-azide to the 5′-DBCO 2′-OMe ASO (Fig. 2b), achieving >90% yields for all bioconjugation reactions performed and >95% purity post HPLC purification (ESI Fig. 1 and 2†). Moreover, to assess off-target effects of the ispinesib moiety itself at the same concentration it will be present in the cell, as part of a covalent-ASO conjugate, we synthesised an ispinesib non-targeting control phosphorothioate ASO (ispinesib-NTC-ASO)^26^ (ESI Fig. 3†). To minimise potential non-specific interactions, we employed a one-step copper-catalysed chemistry for minimal linker effects.

NCL1 transcript levels were measured using reverse transcription-quantitative polymerase chain reaction (RT-qPCR) in HEK293T, A549, and HeLa cells upon lipofectamine transfection at 24 hours, all normalised to the housekeeping gene GAPDH. We compared NCL1 transcript levels upon NCL1 2′-OMe LyTON treatment to the unconjugated NCL1 2′-OMe ASO, 5′-DBCO-modified NCL1 2′-OMe ASO, and the ispinesib-NTC-ASO. The DBCO-modified ASO was used to account for any effects of modifying the terminal with a hydrophobic end group. The NCL1 2′-OMe ASO and DBCO-modified counterpart showed similarly poor knockdown, as they are only able to sterically block the target mRNA (ESI Fig. 4†). However, the NCL1 2′-OMe LyTON significantly outperformed its unconjugated counterpart at all concentrations tested (Fig. 2d, f, h). As expected, for the ispinesib-NTC-ASO, we observed little/no reduction in NCL1 transcript levels (ESI Fig. 4†). Furthermore, we measured little/no differences in the toxicity profiles of the unconjugated and ispinesib-conjugated ASOs, assessed by Cell Titer Glo (ESI Fig. 5–7†) and RNA transcript levels of key housekeeping genes (ESI Fig. 8†). This lines up with our initial expectations, as our treatments are in the sub-micromolar range, well below concentrations that result in toxicity associated with kinesin spindle protein (Eg5) inhibition (the canonical target of ispinesib). Using the SPAAC chemistry, we observed two regioisomer NCL1 2′-OMe LyTON products (ESI Fig. 2†). Both regioisomers exhibited comparable activity, indicating that the isomeric configuration of the ispinisib from the DBCO linkage does not influence the functional activity of the conjugate (ESI Fig. 5†). All further experiments were conducted using the combination of both regioisomers.

After seeing an improvement in knockdown efficiency using transfection, we sought to assess activity in a more clinically relevant manner by carrying out gymnotic delivery of the LyTON conjugate, comparing it to the unconjugated and DBCO-modified counterpart, and the ispinesib-NTC-ASO, at 5, 10, and 20 μM over 96 hours, quantified using RT-qPCR as above. As previously, the ispinesib-NTC-ASO showed little/no knockdown and the DBCO-modified NCL1-2′-OMe ASO gave similar results to the unconjugated NCL1-2′-OMe ASO (ESI Fig. 10†). Whereas, the gymnotically delivered LyTON showed a significant increase in knockdown compared to the unconjugated NCL1-2′-OMe ASO at all the concentrations tested, across all cell types (Fig. 2e, g and i). All together, these results suggest that the ispinesib modification specifically enhances the potency of RNase H-inactive ASOs, without introducing sequence-independent off-target effects.

**Fig. 3:**
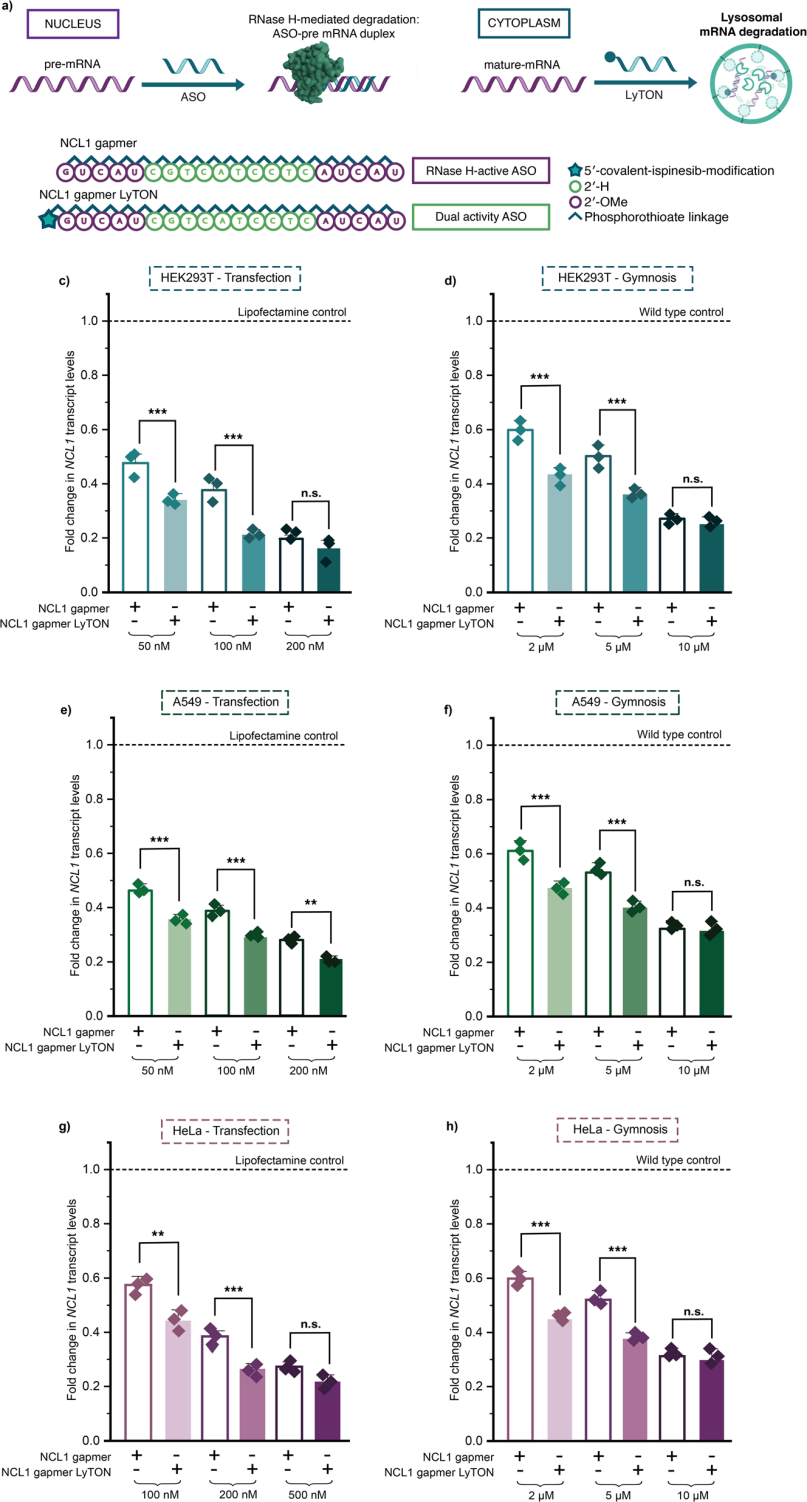
Covalent ispinesib modification of RNase H-active gapmer ASO yields a “dual activity” conjugate with enhanced knockdown capacity of target mRNA. (a) Proposed mechanism of action for the lysosome trafficking-ispinesib ASO (LyTON) conjugates for mRNA knockdown, in contrast to the established RNase H mechanism for mRNA knockdown of ASOs. (b) Sequence and chemical modifications of NCL1 gapmer ASOs used. (c–h) RT-qPCR data for NCL1 knockdown upon lipofectamine transfection (c, e and g – for 24 hours) or gymnotic delivery (e, g and i – for 96 hours) of NCL1 gapmer LyTON and unconjugated-NCL1 gapmer ASO in HEK293T (c and d), A549 (e and f), or HeLa (g and h) cells at concentrations indicated. Three biological replicates in c–h, are shown as diamonds for each condition (each from three technical replicates). The vertical bars represent the mean and the error bars the standard deviation. ** represents *p* < 0.05, *** represents *p* < 0.01, n.s. represents *p* values that are not significant – determined *via* unpaired student's *t*-test.

The NCL1 ASO sequence chosen was originally designed for RNase H-mediated knockdown as a ‘gapmer’. The current state-of-the-art for ASO modification chemistry is the ‘gapmer’ design comprised of wings of five bases containing 2′-methoxyethyl sugars and a central DNA region.^27^ Thus, we sought to explore whether the covalent ispinesib modification of the NCL1 ‘gapmer’ ASO could boost its efficacy. We synthesised a ‘dual activity’ conjugate with ispenisib conjugated to the NCL1 gapmer, harnessing both RNase H-mediated degradation and lysosome trafficking (Fig. 3a and b). Synthesis was carried out as previously, from a 5′-terminally amine-modified gapmer (ESI Fig. 11 and 12†).

The NCL1 gapmer LyTON, along with the unconjugated and 5′-DBCO-modified NCL1 gapmer, were transfected using lipofectamine 2000 into HEK239T, A549 and HeLa cells. NCL1 transcript levels were measured at 24 hours using RT-qPCR. The ‘dual activity’ NCL1 gapmer LyTON outperformed the unmodified and DBCO-NCL1 gapmer at the middle and lower concentrations, in all tested cell lines (Fig. 3c, e, g and ESI Fig. 13†). Using higher concentrations, the activity is significantly improved for A549, but it reached a plateau for HEK293T and HeLa. We then assessed activity *via* gymnotic uptake of the NCL1 gapmer LyTON, compared to the unmodified and DBCO-modified NCL1 gapmer – to served as a more clinically relevant measure of efficacy. As with transfection, using gymnotic delivery we observed a significant increase in the knockdown efficacy of the dual activity, NCL1 gapmer LyTON, compared to the NCL1 gapmer at all but the highest tested concentrations, across all different cell lines (Fig. 3d, f, h and ESI Fig. 14†).

**Fig. 4:**
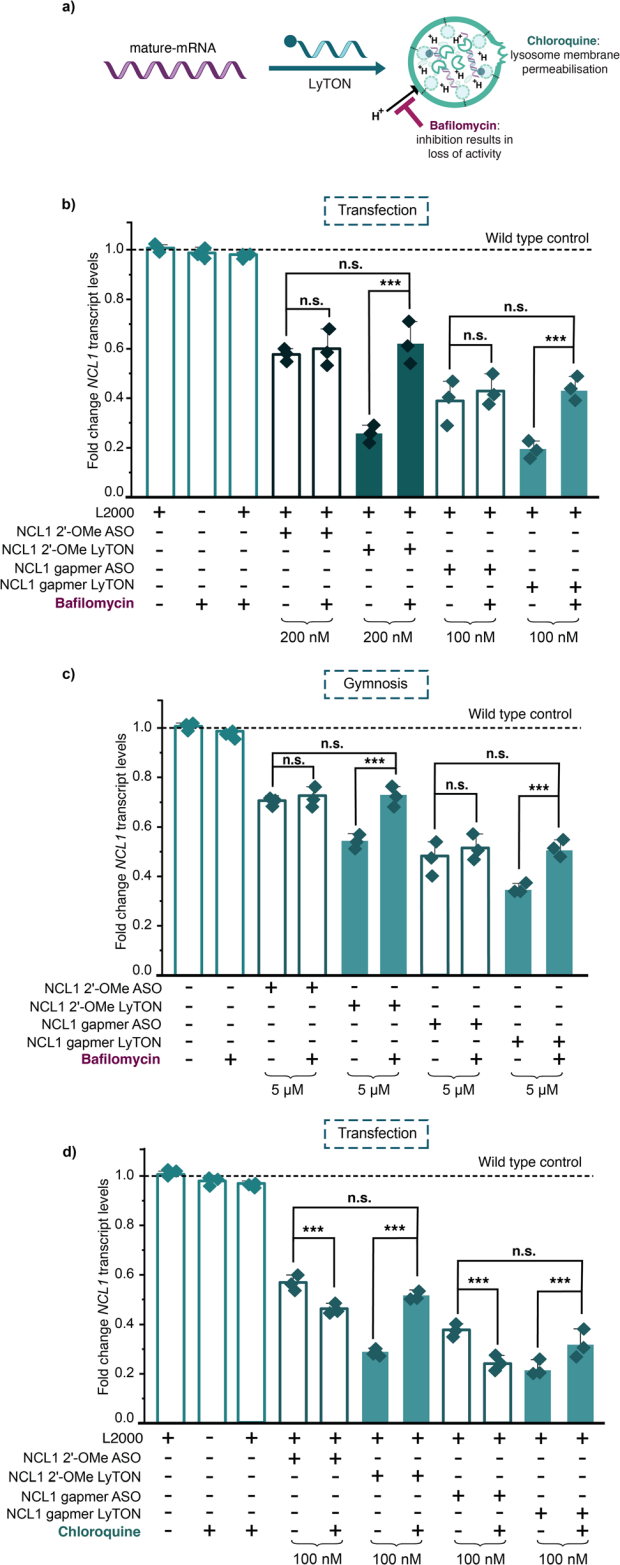
LyTON-warhead ispinesib unlocks a new mechanism of ASO-mediated mRNA degradation *via* lysosomal trafficking, validated using bafilomycin and chloroquine. (a) Mechanism of bafilomycin-mediated lysosomal inhibition in relation to LyTON activity. (b) RT-qPCR data for NCL1 knockdown upon lipofectamine transfection of NCL1 gapmer ASO, NCL1 gapmer LyTON, NCL1 2′-OMe ASO, and NCL1 2′-OMe LyTON in HEK293T cells in the presence or absence of 10 nM bafilomycin, at the concentrations indicated. (c) RT-qPCR data for NCL1 knockdown upon gymnosis of NCL1 gapmer ASO, NCL1 gapmer LyTON, NCL1 2′-OMe ASO, and NCL1 2′-OMe LyTON in HEK293T cells in the presence or absence of 10 nM bafilomycin, at the concentrations indicated. (d) RT-qPCR data for NCL1 knockdown upon lipofectamine transfection of NCL1 gapmer ASO, NCL1 gapmer LyTON, NCL1 2′-OMe ASO, and NCL1 2′-OMe LyTON in HEK293T cells in the presence or absence of 30 μM chloroquine, at the concentrations indicated. Three biological replicates are shown as diamonds for each condition (each from three technical replicates). The vertical bars represent the mean and the error bars the standard deviation. ** represents *p* < 0.05, *** represents *p* < 0.01, n.s. represents *p* values that are not significant – determined *via* unpaired student's *t*-test.

Our results demonstrated that we could achieve a significant increase in reduction of target mRNA when conjugating ispinesib to NCL1 RNase H-inactive or RNase H-active ASOs. Next, we sought to confirm whether the mechanism of mRNA degradation involved the lysosome, using inhibitors of various aspects of lysosomal function: bafilomycin and chloroquine (Fig. 4a).

Bafilomycin is a well-characterised inhibitor of lysosome activity^28,29^ (Fig. 4a). Bafilomycin inhibits vacuolar V-ATPase and prevents lysosomal acidification – disrupting basal lysosomal flux. If the LyTONs were trafficking the target mRNA to the lysosome, treatment with bafilomycin would result in loss of knockdown activity (Fig. 4a). First, we treated the HEK293T cells with bafilomycin for 24 h and confirmed the inhibition of lysosomal activity through an increase in LC3-II levels (ESI Fig. 15†). Since cell toxicity was observed with 100 nM bafilomycin, which could lead to an inaccurate assessment of ASO efficacy, the lower 10 nM concentration (without any cell toxicity effects) was chosen for further studies with the ASOs (ESI Fig. 16†). The knockdown of NCL1 transcript levels was then measured upon transfection with the NCL1 2′-OMe ASO, NCL1 2′-OMe LyTON, NCL1 gapmer, and NCL1 gapmer LyTON in the presence and absence of bafilomycin (Fig. 4b). As expected, the bafilomycin treatment did not affect the activity of the NCL1 2′-OMe ASO or NCL1 gapmer. However, the enhanced mRNA knockdown activity of both the NCL1 2′-OMe LyTON and NCL1 gapmer LyTON were completely lost in the presence of bafilomycin, showing comparable activity to the parent unmodified NCL1 2′-OMe ASO or NCL1 gapmer, respectively. We observed the same trend for Bafilomycin inhibition upon lipofectamine transfection in A549 (ESI Fig. 17†) and HeLa cells (ESI Fig. 18†).

To eliminate any confounding effects of lipofectamine-mediated transfection, we carried out the same bafilomycin inhibition assay using gymnotic delivery of NCL1 2′-OMe ASO, NCL1 2′-OMe LyTON, NCL1 gapmer, and NCL1 gapmer LyTON (Fig. 4c). We observed the same trend – the bafilomycin treatment did not affect the activity of the NCL1 2′-OMe ASO or NCL1 gapmer upon gymnotic delivery. However, the increased mRNA knockdown activity of both the NCL1 2′-OMe LyTON and NCL1 gapmer LyTON, when delivered gymnotically, were completely lost in the presence of bafilomycin, with activity restored to the parent unmodified NCL1 2′-OMe ASO or NCL1 gapmer, respectively. This confirmed that LyTONs relied upon lysosomal degradation for activity, and upon inhibition of normal lysosomal flux only acted as a conventional ASOs.

Chloroquine is another well-characterised inhibitor of lysosomal activity – known to accumulate in the lysosome due to its weakly basic nature, raising intra-lysosomal pH. This leads to lysosomal membrane permeabilization causing partial leakage of lysosomal contents into the cytosol (Fig. 4a).^30^ Due to this, chloroquine has been widely used to increase the endosomal/lysosomal escape of ASOs to increase their activity within cells.^31^ We first conducted a dose titration as excessive chloroquine can cause broad lysosomal dysfunction and cytotoxicity, which may in turn obscure or diminish ASO activity (ESI Fig. 19†). We then transfected the NCL1 gapmer and NCL1 2′-OMe ASO in the presence and absence of 30 μM chloroquine and, as expected from improved endosomal/lysosomal escape, observed a significant improvement in the activity of both conventional ASOs (Fig. 4d). In contrast, the chloroquine treatment inhibited the activity of both the NCL1 2′-OMe LyTON and NCL1 gapmer LyTON (Fig. 4d). This confirmed that functional lysosomal integrity is necessary for the LyTON mechanism of action. These findings highlight a key distinction in the intracellular processing pathways between unconjugated ASOs, which may benefit from disrupted lysosomal barriers, and LyTON conjugates, which require intact lysosomal processing machinery for activity.

After establishing the chemistry and mechanism of action of LyTONs, we decided to target an exciting therapeutically-relevant gene of interest. For this, we chose the MEN1 gene that encodes for the protein, Menin – which plays a particularly significant role in MLL-rearranged leukemias. Menin acts as a scaffold for oncogenic MLL fusion proteins resulting in the transcriptional activation programs for leukemic cell survival and growth.^32^ Menin also has a key role in KRAS-driven cancers.^33^ Inhibition of Menin has been shown to disrupt such cancer–sustenance pathways, leading to reduced proliferation and increased apoptosis.^34,35^ Small-molecule Menin inhibitors have shown early clinical promise, however, recent clinical studies have found emerging resistance and patient relapse due to somatic mutations in MEN1.^36^ The structural mutations in Menin occur within a key region of the inhibitor-binding pocket essential for stabilizing Menin inhibitor binding but not required for interaction with MLL. These small molecule drug resistance challenges, which manifest at the protein level, can potentially be circumvented by targeting MEN1 mRNA using ASOs.

**Fig. 5:**
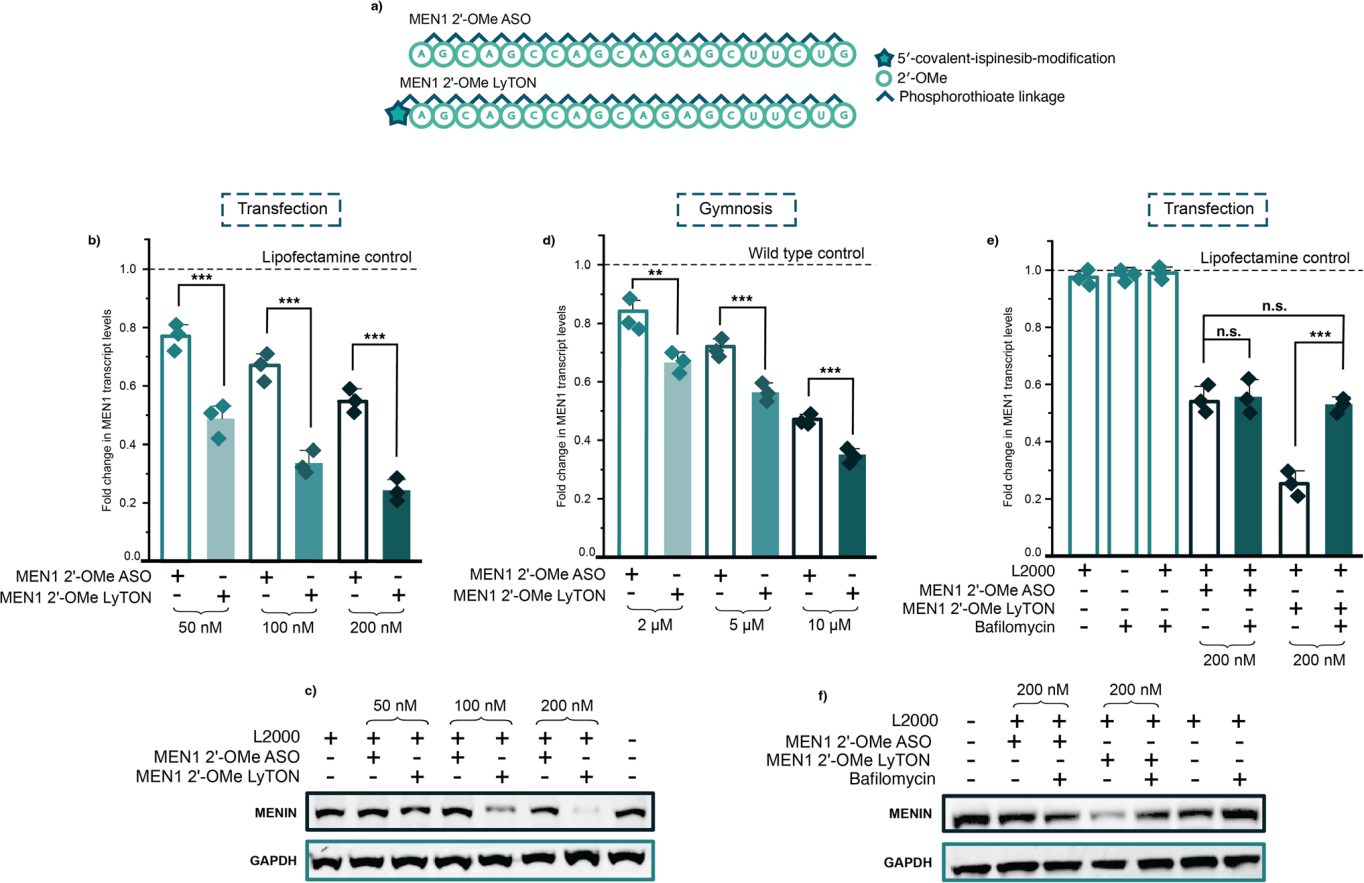
MEN1 2′-OMe LyTON treatment results in significant knockdown of MEN1 mRNA and protein. (a) Sequence and chemical modifications of ASOs used to target MEN1. (b) RT-qPCR data and (c) western blot for MEN1/Menin knockdown upon lipofectamine transfection of MEN1 2′-OMe ASO and MEN1 2′-OMe LyTON ASO in HEK293T cells at concentrations indicated. (d) RT-qPCR data for MEN1/Menin knockdown upon gymnosis of MEN1 2′-OMe ASO and MEN1 2′-OMe LyTON ASO in HEK293T cells at the concentrations indicated for 96 hours. (e) RT-qPCR data and (f) western blot for MEN1/Menin knockdown upon lipofectamine transfection of MEN1 2′-OMe ASO and MEN1 2′-OMe LyTON ASO in HEK293T cells in the presence or absence of 10 nM bafilomycin, at the concentrations indicated. Three biological replicates in (b), (d), and (e) are shown as diamonds for each condition (each from three technical replicates). The vertical bars represent the mean and the error bars the standard deviation. ** represents *p* < 0.05, *** represents *p* < 0.01, n.s. represents *p* values that are not significant – determined *via* unpaired student's *t*-test.

A well-established MEN1 ASO sequence was chosen and purchased containing all 2′-OMe-modified sugars (RNase H-inactive)^37^ (Fig. 5a). As with the NCL1 LyTONs, ispinesib was conjugated using SPAAC chemistry on the 5′-terminus (ESI Fig. 20 and 21†). MEN1 transcript levels were measured after transfection of the MEN1 2′-OMe LyTON using RT-qPCR in HEK293T cells at 24 hours, comparing the knockdown to the unconjugated and 5′-DBCO-modified MEN1 2′-OMe ASO, and the ispinesib-NTC-ASO, all normalised to the housekeeping gene GAPDH. As previously, the ispinesib-NTC-ASO showed little/no knockdown and the DBCO-modified MEN1 2′-OMe ASO gave similar results to the unconjugated MEN1 2′-OMe ASO (ESI Fig. 22†). In contrast, the MEN1 2′-OMe LyTON significantly outperformed the unmodified MEN1 2′-OMe ASO at all concentrations tested (Fig. 5b). Futhermore, we observed the same trends in A549 and HeLa (ESI Fig. 23 and 24†) cells, at all tested concentrations. We also observed a marked reduction in Menin protein levels upon MEN1 2′-OMe LyTON treatment, compared to the unconjugated ASO, in a concentration dependant manner (Fig. 5c and ESI Fig. 25†). To further assess specificity, we tested each ispinesib-conjugate for off-target effects by evaluating knockdown of the alternate ASO target used in this study in HEK293T, A549, and HeLa cells. Specifically, we observed that the NCL1 2′-OMe and gapmer LyTONs did not reduce MEN1 transcript levels (ESI Fig. 26†), and conversely, the MEN1 2′-OMe and gapmer LyTONs did not impact NCL1 expression (ESI Fig. 27†): confirming target-specific knockdown.

The MEN1 2′-OMe LyTON also exhibited improved knockdown when delivered gymnotically. This effect was observed across all tested concentrations in HEK293T cells after gymnosis for 96 hours, and assessed using RT-qPCR (Fig. 5d and ESI Fig. 28†). The same trend for higher efficacy using the LyTON counterpart, compared to the unconjugated and DBCO-modified ASO, was also observed using gymnotic delivery in A549 and HeLa cells (ESI Fig. 29 and 30†). We then sought to again confirm the mechanism of action for the ispinesib-ASO conjugates, using bafilomycin treatment. As with the NCL1 LyTON, treatment with bafilomycin resulted in the complete loss of enhanced mRNA knockdown for the MEN1 2′-OMe LyTON (Fig. 5e). Only steric blocking activity on par with the parent MEN1 2′-OMe ASO was observed. This loss of activity upon treatment with bafilomycin was also observed at the protein level (Fig. 5f and ESI Fig. 31†). Additionally, we observed little/no differences in the toxicity profiles of the unconjugated and ispinesib-conjugated MEN1 ASOs, as previously, assessed through Cell Titer Glo in all tested cell lines (ESI Fig. 32–34†) and transcript levels of key housekeeping genes (ESI Fig. 35†).

## Discussion/future perspectives

Targeted mRNA degradation is a powerful strategy for therapeutic intervention: targeting the “undruggable”, allowing for personalised medicine with “*n*-of-1” therapies, and, potentially, circumventing traditional small molecule drug resistance mechanisms. Currently, mRNA knockdown can only be achieved through RNase H recognition of DNA-based ASOs or RNA-induced silencing complex (RISC) formation with siRNA. Our work introduces an entirely new mechanism for targeted mRNA degradation. By conjugating ispinesib, an ATTEC warhead, to ASOs, target mRNA is trafficked to the lysosome for subsequent degradation. These lysosomal trafficking antisense oligonucleotides (LyTONs) are modular and versatile. These conjugates can be generated through simple post-synthetic modification chemistry in high yields with high purity. LyTONs demonstrate improved knockdown efficacy across various molecular targets and cell lines, utilizing both lipofectamine and gymnotic delivery. Furthermore, by combining the mechanisms of action of lysosomal trafficking and RNase H, LyTONs boost the activity of state-of-the-art gapmer ASOs. The therapeutic potential of LyTONs is reflected in the targeting of Menin, an exciting target in cancer medicine with effective knockdown at the protein level.

RNase H-active ASOs are predominantly functional in the nucleus, as this is where the highest levels of RNase H preside. However, mRNA is shuttled out of the nucleus for translation. While siRNA carry out cytoplasmic degradation of mRNA, they are even more difficult to deliver into cells than ASOs.^38,39^ Our approach leverages a lysosomal degradation pathway that improves target engagement, and thus, can potentially enable therapeutic targeting of more clinically-relevant genes using ASOs. Using our LyTON technology, we will be able to rethink the design of gene-silencing oligonucleotides. Without the need for incorporating DNA-containing sequences for RNase H recognition or RNA-containing sequences for RISC formation, many alternative oligonucleotide chemistries could be explored for nucleic acid drug development-allowing for ASO chemistries with higher chemical stability, higher target affinity, and improved delivery profiles for gene knockdown applications. A key future goal will be to fully characterise the binding partner of ispinesib for lysosomal trafficking.^40–43^ This should enable the development of improved LyTONs through rational ligand design.

In summary, our work represents a significant advancement in the field of targeted mRNA degradation. By harnessing lysosomal trafficking through the design of a bi-functional ispinesib-ASO conjugate, we offer a powerful new strategy for selective mRNA degradation. This approach holds great promise in precision medicine, particularly in expanding the use of diverse oligonucleotide chemistries for gene silencing. With its modular design and capacity for future advancements, this technology could emerge as a powerful platform for nucleic acid-based therapeutic strategies.

## Author contributions

D. K., T. A. M., and M. J. B. designed the project. D. K. designed, performed, and analysed the experiments, with contributions from T. M. and M. J. B. All authors wrote the paper.

## Conflicts of interest

T. A. M. is a shareholder and consultant for Dark Blue Therapeutics. D. K. and M. J. B. declare no conflict of interest.

## Supplementary Material

SC-OLF-D5SC03751D-s001

## Data Availability

All the data generated in this study are available within the article, the ESI,† and figures. Source data are available from DOI: https://doi.org/10.5281/zenodo.15685343.
