# Supplementary material for: Engineering antisense oligonucleotides for targeted mRNA degradation through lysosomal trafficking
Source: Chem Sci. 2025 Jun 9;16(28):13096–105. doi: 10.1039/d5sc03751d (PMC12180356; doi:10.1039/d5sc03751d)
Supplement: SC-016-D5SC03751D-s001 [file SC-016-D5SC03751D-s001.pdf]

Supplementary Information (SI) for Chemical Science.
This journal is © The Royal Society of Chemistry 2025

Supplementary Information for:

Engineering antisense oligonucleotides for targeted mRNA degradation through lysosomal trafficking

Disha Kashyap[1,2], Thomas A. Milne[2,*], Michael J. Booth[1,3,*]

[1]Department of Chemistry, University of Oxford, Mansfield Road, Oxford, OX1 3TA, U.K.
[2]MRC Molecular Haematology Unit, MRC Weatherall Institute of Molecular Medicine, Radcliffe Department of Medicine, University of Oxford, Oxford, OX3 9DS, UK
[3]Department of Chemistry, University College London, 20 Gordon Street, London, WC1H 0AJ, U.K.

*Correspondence: thomas.milne@imm.ox.ac.uk, m.j.booth@ucl.ac.uk

Table of Contents

1. **Materials and Methods**……………………………………………………………………………….........2
   1.1. Nucleic Acid Chemistry Functionalisation, Purification, and Characterisation
      1.1.1. Dibenzylcyclooctyne (DBCO)-NHS ester functionalisation
      1.1.2. Ispinesib-functionalisation with strain-promoted azide-alkyne click chemistry
      1.1.3. Ispinesib-functionalisation with copper-catalysed click chemistry
      1.1.4. Oligonucleotide MS characterisation

2. **Nucleic Acid Sequences**………………………………………………………………………………...4
   2.1. Table 1: Antisense oligonucleotide sequences used
   2.2. Table 2: Molecular weights for oligonucleotide conjugates prepared

3. **Biological Assays**……………………………………………………………………………………......4
   3.1. Cell Culture
   3.2. Transfection
   3.3 Gymnosis
   3.4 Cell Titer-Glo
   3.5 Lysosome inhibition assays
      3.5.1 Bafilomycin inhibition
      3.5.2 Chloroquine inhibition
   3.6. RT-qPCR
      3.6.1. Table 3: qPCR primers sequences
   3.7. Western Blotting

4. Supplementary Figures……………………………………………………………………………………7
4.1. Figure S1: Reaction and characterisation for DBCO modification of NCL1 2'-OMe ASO.
4.2. Figure S2: Reaction and characterisation for ispinesib modification of NCL1 2'-OMe ASO.
4.3. Figure S3: Reaction and characterisation for ispinesib modification of NTC-ASO.
4.4. Figure S4: RT-qPCR data of NCL1 knockdown upon DBCO-modified NCL1 2'-OMe ASO and ispinesib-NTC-ASO lipofectamine transfection in a) HEK293Ts, b) A549, c) HeLa for 24 hours at concentrations indicated.
4.5. Figure S5: Cell viability of HEK293T cells upon NCL1 2'-OMe ASO and NCL1 2'-OMe LyTON treatment evaluated by Cell-Titer Glo.
4.6. Figure S6: Cell viability of A549 cells upon NCL1 2'-OMe ASO and NCL1 2'-OMe LyTON treatment evaluated by Cell-Titer Glo.
4.7. Figure S7: Cell viability of HeLa cells upon NCL1 2'-OMe ASO and NCL1 2'-OMe LyTON treatment evaluated by Cell-Titer Glo.
4.8. Figure S8: Ct values for key housekeeping genes upon NCL1 2'-OMe ASO and NCL1 2'-OMe LyTON treatment at 200 nM in HEK293T.

4.9. Figure S9: LC-MS characterisation and RT-qPCR for regioisomers for ispinesib modification of NCL1 2'-OMe ASO.
4.10. Figure S10: RT-qPCR data of NCL1 knockdown upon unmodified NCL1 2'-OMe ASO, DBCO-modified NCL1 2'-OMe ASO and ispinesib-NTC-ASO gymnosis in a) HEK293Ts, b) A549, c) HeLa for 96 hours at concentrations indicated.
4.11. Figure S11: Reaction and characterisation for DBCO modification of NCL1 gapmer ASO.
4.12. Figure S12: Reaction and characterisation for ispinesib modification of NCL1 gapmer ASO.
4.13. Figure S13: RT-qPCR data of NCL1 knockdown upon unmodified and DBCO-modified NCL1 gapmer lipofectamine transfection in a) HEK293Ts, b) A549, c) HeLa for 24 hours at concentrations indicated.
4.14. Figure S14: RT-qPCR data of NCL1 knockdown upon unmodified and DBCO-modified NCL1 gapmer gymnosis in a) HEK293Ts, b) A549, c) HeLa for 96 hours at concentrations indicated.
4.15. Figure S15: Uncropped western for LC3-I and LC3-II levels upon treatment with bafilomycin at indicated concentrations and time points, in HEK293Ts
4.16. Figure S16: Cell viability upon bafilomycin (Bafa) treatment in HEK293Ts assayed by dye exclusion on hemocytometer.
4.17. Figure S17: RT-qPCR data for NCL1 knockdown upon lipofectamine transfection with NCL1 gapmer ASO, NCL1 gapmer LyTON, NCL1 2'-OMe ASO, and NCL1 2'-OMe LyTON in A549 cells in the presence or absence of 10 nM bafilomycin, at the concentrations indicated.
4.18. Figure S18: RT-qPCR data for NCL1 knockdown upon lipofectamine transfection of NCL1 gapmer ASO, NCL1 gapmer LyTON, NCL1 2'-OMe ASO, and NCL1 2'-OMe LyTON in HeLa cells in the presence or absence of 10 nM bafilomycin, at the concentrations indicated.
4.19. Figure S19: Cell viability upon chloroquine (CQ) treatment in HEK293Ts assayed by dye exclusion on hemocytometer.
4.20. Figure S20: Reaction and characterisation for DBCO modification of MEN1 2'-OMe ASO.
4.21. Figure S21: Reaction and characterisation for ispinesib modification of MEN1 2'-OMe ASO.
4.22. Figure S22: RT-qPCR data of MEN1 knockdown upon unmodified MEN1 2'-OMe ASO, DBCO-modified MEN1 2'-OMe ASO and ispinesib-NTC-ASO lipofectamine transfection in HEK293Ts for 24 hours at concentrations indicated.
4.23. Figure S23: RT-qPCR data of MEN1 knockdown upon unmodified MEN1 2'-OMe ASO, DBCO-modified MEN1 2'-OMe ASO and ispinesib-NTC-ASO lipofectamine transfection in a) A549, b) HeLa for 24 hours at concentrations indicated.
4.24. Figure S24. RT-qPCR data for MEN1 knockdown upon lipofectamine transfection of unmodified MEN1 2'-OMe ASO and MEN1 2'-OMe LyTON ASO in a) A549, b) HeLa at the concentrations indicated for 24 hours.
4.25. Figure S25: Uncropped western blot of Menin levels upon treatment with MEN1 2'-OMe ASO and MEN1 2'-OMe LyTON upon transfection with lipofectamine, harvested at 48 hours in HEK293Ts.
4.26. Figure S26. RT-qPCR data of MEN1 knockdown upon NCL1 2'-OMe LyTON lipofectamine transfection in a) HEK293T, b) A549, c) HeLa for 24 hours at concentration indicated.
4.27. Figure S27. RT-qPCR data of NCL1 knockdown upon MEN1 2'-OMe LyTON lipofectamine transfection in a) HEK293T, b) A549, c) HeLa for 24 hours at concentration indicated.
4.28. Figure S28: RT-qPCR data of MEN1 knockdown upon DBCO-modified MEN1 2'-OMe ASO and ispinesib-NTC-ASO gymnosis in HEK293Ts for 96 hours at concentrations indicated.
4.29. Figure S29: RT-qPCR data of MEN1 knockdown upon DBCO-modified MEN1 2'-OMe ASO and ispinesib-NTC-ASO gymnosis in a) A549, b) HeLa for 96 hours at concentrations indicated.
4.30. Figure S30: RT-qPCR data of MEN1 knockdown upon unmodified MEN1 2'-OMe ASO and MEN1 2'-OMe LyTON ASO gymnosis in a) A549, b) HeLa for 96 hours at concentrations indicated.
4.31. Figure S31: Uncropped western blot of Menin levels upon treatment with MEN1 2'-OMe ASO and MEN1 2'-OMe LyTON – in presence and absence of 10 nM bafilomycin, upon transfection with lipofectamine, harvested at 48 hours in HEK293Ts.
4.32. Figure S32: Viability of HEK293T cells upon MEN1 2'-OMe ASO and MEN1 2'-OMe LyTON treatment evaluated by Cell-Titer Glo.
4.33. Figure S33: Viability of A549 cells upon MEN1 2'-OMe ASO and MEN1 2'-OMe LyTON treatment evaluated by Cell-Titer Glo.

4.34. Figure S34: Viability of HeLa cells upon MEN1 2'-OMe ASO and MEN1 2'-OMe LyTON treatment evaluated by Cell-Titer Glo.
4.35. Figure S35: Ct values for key housekeeping genes upon MEN1 2'-OMe ASO and MEN1 2'-OMe LyTON treatment at 200 nM.

5. References..........28

1. Materials and Methods

1.1 Nucleic acid chemistry functionalisation, purification, and characterisation

1.1.1 Dibenzylcyclooctyne (DBCO)-NHS ester functionalisation

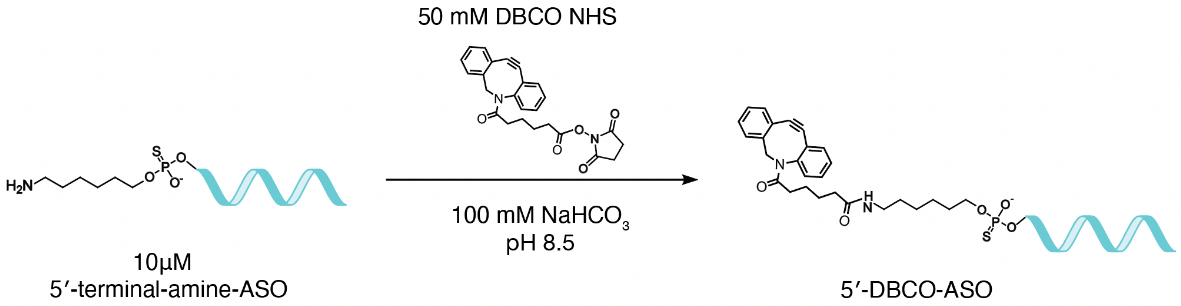

To a 0.5 mL Eppendorf DNA LoBind tube was added 1 µL of the DNA (100 µM stock concentration), 1 µL of $NaHCO_3$ pH 8.5 (1 M), 2 µL of H_2O, 5 µL of a 50 mM solution of DBCO NHS in dry DMF. The reaction was vortexed, spun down in a tabletop centrifuge and placed in a Thermomixer (Eppendorf) overnight, shaking at 800 rpm at 37°C.

The excess hydrolysed small molecule was removed using Amicon 3K 0.5 mL spin columns, as per the manufacturer's instructions. Briefly, the reaction was made up to a volume of 500 µL with H_2O and spun at 14,700g for 10 minutes. The eluent was removed, and the above process was repeated for a total of three times. The column was then inverted, inserted into a fresh collection tube and spun at 14,700g for 1 minute. The eluent obtained was then purified by HPLC on an Agilent Polaris C18 column (150 x 4.6 mm), column heated to 50°C using a gradient of 3-30% CH_3CN (indicated in **SI** figures) over 20 minutes, flow rate of 1.5 mL/min, with 10 mM triethylammonium bicarbonate (TEAB) pH 8.5 as an ion-pairing buffer throughout.

1.1.2 Ispinesib-functionalisation with strain-promoted azide-alkyne click chemistry

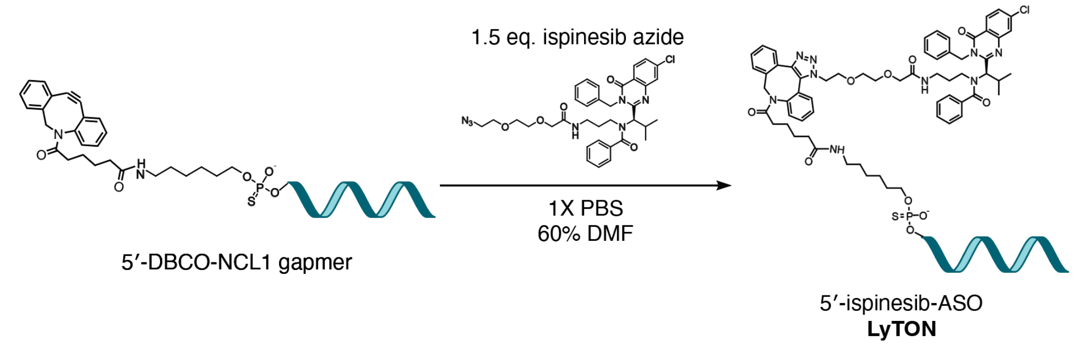

To a 0.5 mL Eppendorf DNA LoBind tube was added, listed in order of addition, 1 μL of the DNA (1 mM stock concentration), 1.5 μL of 1 mM ispinesib-azide (commercially obtained from MedChemExpress), 1.5 μL of 1X PBS pH 7.4 and finally, 6 μL of DMF. The reaction was vortexed, spun down in a tabletop centrifuge and placed in a Thermomixer (Eppendorf) overnight, shaking at 800 rpm at room temperature.

The crude reaction mixture was then made up to 500 μL with H_2O and first run through a Amicon 3K 0.5 mL column/tube as described above to remove the unreacted small molecule. The final eluent collected after inversion was then purified by HPLC on an Agilent Polaris C18 column (150 x 4.6 mm), column heated to 50°C using a gradient of 3-50% CH_3CN (indicated in **SI** figures) over 20 minutes, flow rate of 1.5 mL/min, with 10 mM triethylammonium bicarbonate (TEAB) pH 8.5 as an ion-pairing buffer throughout.

1.1.3 Ispinesib-functionalisation with copper-catalysed click chemistry

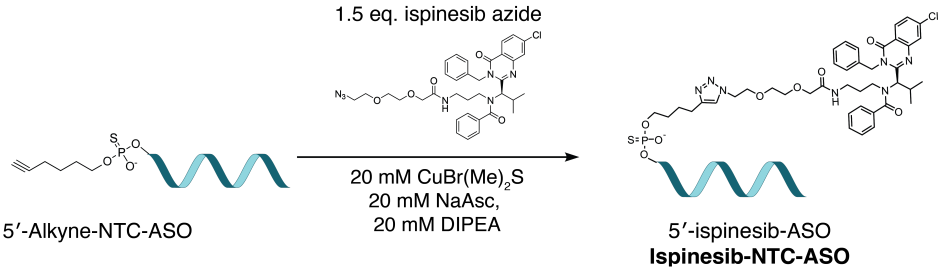

To a 0.5 mL Eppendorf DNA LoBind tube was added, listed in order of addition, 1 μL of the alkyne-containing PS-modified DNA NTC-ASO (1 mM stock concentration), 1.5 μL of 1 mM ispinesib-azide, molecule **2**, 1 uL of 200 mM DIPEA, 5.5 μL of H_2O, 1 μL of 200 mM sodium ascorbate and finally, 1 μL of 200 mM copper (I) bromide-dimethyl sulphide. The reaction was vortexed, spun down in a tabletop centrifuge and placed in a Thermomixer (Eppendorf) overnight, shaking at 800 rpm at room temperature.

The reaction was quenched with 250 μL of 0.5 M EDTA pH 8, made up to 500 μL with H_2O and first run through a Amicon 3K 0.5 mL column/tube as described above to chelate excess copper and remove it along with the unreacted small molecule. The final eluent collected after inversion was then purified by HPLC on an Agilent Polaris C18 column (150 x 4.6 mm), column heated to 50°C using a gradient of 3-50% CH_3CN (indicated in **SI** figures) over 20 minutes, flow rate of 1.5 mL/min, with 10 mM triethylammonium bicarbonate (TEAB) pH 8.5 as an ion-pairing buffer throughout.

1.1.3 Oligonucleotide MS characterisation

Oligonucleotide Mass Spectra were recorded on a Waters Xevo G2 QTOF ESI-UPLC-MS system. A gradient of MeOH in Et_3N and hexafluoroisopropanol (HFIP) was used (buffer A, 8.6 mM Et_3N, 200 mM HFIP in 5% MeOH/H_2O (v/v); buffer B, 20% buffer A in MeOH). Data was then deconvoluted using MassLynx software v4.1 and recorded in **Table 2**.

2. Nucleic acid sequences

qPCR primers (unmodified oligonucleotides) were purchased from IDT as desalted and lyophilised products – resuspended in 10 mM Tris pH 8. All ASOs (modified and amine-, and alkyne-containing modified oligonucleotides) were purchased from IDT, HPLC-purified in lyophilised form and dissolved in 10 mM potassium phosphate buffer, pH 8.

2.1 Table 1: Antisense oligonucleotide sequences used for all transfection experiments with 5′-terminal amine modifiers for aforementioned functionalisation chemistry

* denotes phosphorothioate linkages

_ denotes 2′-Methoxy-ethyl sugars

m denotes 2′-O-Methyl sugars

No.	Name	Sequence (5'-3')
1	NCL1 2'-OMe ASO	mG*mU*mC*mA*mU*mC*mG*mU*mC*mA*mU*mC*mC*mU*mC*mA*mU*mC*mA*mU
2	NCL1 gapmer	G*T*C*A*T*C*G*T*C*A*T*C*C*T*C*A*T*C*A*T
3	MEN1 2'-OMe ASO	mA*mG*mC*mA*mG*mC*mC*mA*mG*mC*mA*mG*mA*mG*mC*mU*mU*mC*mU*mG
4	MEN1 gapmer	A*G*C*A*G*C*C*A*G*C*A*G*A*G*C*U*U*C*U*G
5	NTC-ASO	T*A*G*T*G*C*G*G*A*C*C*T*A*C*C*C*A*C*G*A

2.2 Table 2: Molecular weights for oligonucleotide conjugates prepared

No.	Name	Expected mass	Mass after deconvolution
1	DBCO-NCL1 2'-OMe ASO	7442.0	**7442.7**
2	NCL1 2'-OMe LyTON	8131.0	**8131.5**
3	Ispinesib-NTC-ASO	7240.0	**7239.0**
3	DBCO-NCL1 gapmer	7542.0	**7541.7**
4	NCL1 gapmer LyTON	8231.0	**8231.7**
5	DBCO-MEN1 2'-OMe ASO	7308.0	**7308.6**
6	MEN1 2'-OMe LyTON	7997.0	**7994.6**

3. Biological assays

3.1 Cell culture

HEK293T, A549, and HeLa cells were cultured in Dulbecco’s Modified Eagle Medium with 1X GlutaMAX (Gibco) supplemented with 10% (v/v) FBS (Gibco) and) at 37 °C in a humidified incubator with 5% CO_2.

3.2 Transfection

For transfection with lipofectamine 2000, HEK293T / A549 / HeLa cells were seeded at 2.5×10^5 / 2.2×10^5 / 2.0×10^5 cells in 1 mL of culture media in 24-well plates 16 hours before transfection to reach 70-80% cell confluency. Prior to transfection, the culture media was replaced with 1 mL OptiMEM.

2 µL of Lipofectamine 2000 (Invitrogen) was added to 48 µL OptiMEM (Gibco) and incubated at room temperature for 5 minutes before mixing with 0.5 nmol of oligonucleotide dissolved in 50 µL of OptiMEM. The resulting mixture was incubated at room temperature for 20 minutes allowing complexation to occur. The complexes were then added to the cells at the required concentrations (with total volume of 1 mL per well). The cells were then incubated at 37 °C in a 5% CO_2 incubator. After 6 hours the media was replaced with 1 mL of culture media and the cells were returned to the incubator for another 18 hours.

3.3 Gymnosis

HEK293T / A549 / HeLa cells were seeded at a density of 5.0×10^4 / 4.7×10^4 / 4.5×10^4 cells/well in 1 mL of culture media in 24-well plates for 16 hours before transfection to reach 70-80% cell confluency. Prior to transfection, the culture media was replaced with OptiMEM and the ASO was added at the desired concentration. After 6 hours, OptiMEM was replaced with 1 mL of complete culture media and the cells were returned to the incubator for a further 90 hours (total time of 96 hours).

3.4 CellTiter-Glo

Transfection experiments were carried out as outlined above. Cells were subjected to the CellTiter-Glo assay at the indicated time points following the manufacturer's guidelines. Briefly, 100 µL CellTiter-Glo Reagent was added to 100 µL of media containing cells in a white (Thermofischer Nunc MicroWell polystyrene, 236105) 96-well plate. Subsequently, the contents of the wells were mixed on an orbital shaker for 2 minutes to induce cell lysis. The plate was then incubated at room temperature for 10 minutes to stabilize the luminescent signal. The luminescence was measured using a CLARIOstar microplate reader (BMG Labtech). The luminescence values were normalised to the values for untreated cells.

3.5 Lysosome inhibition assays

3.5.1 Bafilomycin inhibition

Cell were plated at the above mentioned densities and transfection/gymnosis was carried out as above. Bafilomycin was added at 10 nM at the start of the transfection. When the OptiMEM was replaced with complete media, the 10 nM Bafilomycin treatment was repeated. Cells were then harvested at 24 hours, and subjected to RNA extraction/western blotting for RNA/protein read-out.

3.5.1 Chloroquine inhibition

Cell were plated at the above mentioned densities and transfection/gymnosis was carried out as above. When the OptiMEM was replaced with complete media at 6 hours, cells were treated with 30 µM Chloroquine. Cells were then harvested at 24 hours (with 18 hours of chloroquine treatment), and subjected to RNA extraction for RNA read-out.

3.6 RT-qPCR

Total RNA was extracted and DNase I-treated from pellets with 1 × 10^6 cells using the RNeasy Mini kit (Qiagen). RNA was reverse-transcribed using SuperScript III (ThermoFisher Scientific) with random hexamer primers, and then quantified using SYBR Green in QuantStudio 3 Real-time PCR machine (qPCR primers listed in Table 3). Gene expression was normalized to mature mRNA levels of the housekeeping gene, GAPDH. The data is presented as vertical bars which represent the mean and the error bars represent the standard deviation. Difference between lipofectamine and ASO treatment were analysed using unpaired student's t-test.

3.6.1 Table 3: qPCR primer sequences, using SYBR for knockdown quantification

S. No.	Name	Sequence (5'-3')
1	GAPDH FWD	TTGGCTACAGCAACAGGGTG
2	GAPDH REV	GGGGAGATTCAGTGTGGTGG
3	NCL1 FWD	GCCTGTCAAAGAAGCACCTGG
4	NCL1 REV	GAAAGCCGTAGTCGGTTCTGTG
5	MEN1 FWD	ATCGGGCCCATCCAGTCCC
6	MEN1 REV	GCCCCAACCACAGCAAAGGC
7	YWHAZ FWD	ACTTTTGGTACATTGTGGCTTCAA
8	YWHAZ REV	CCGCCAGGACAAACCAGTAT
9	UBC FWD	ATTTGGGTCGCGGTTCTTG
10	UBC REV	TGCCTTGACATTCTCGATGGT

3.7 Western blotting

Salt-soluble proteins were extracted from 1 × 10^6 cells by incubating cells in a high-salt lysis buffer (20 mM Tris-HCl pH 8.0, 300 mM KCl, 5 mM EDTA, 20% glycerol, 0.5% IGEPAL CA-630, protease inhibitor cocktail). Protein extracts were then run on a NuPAGE 4-12% BisTris gels (Life Technologies) for Menin and NuPAGE 12% BisTris gels for LC3 at 180V for 1 hour and blotted onto a polyvinylidene fluoride membrane (Immobilon) at 100V for 1 hour using a Tris-glycine blotting buffer.[1] The blots were then probed with 1:10,000 dilution of primary antibody for Menin (Bethyl #A300-105A) and LC3-I and LC3-II were probed with 1:10,000 dilution of primary antibody for LC3 (Sigma Aldrich, L8918) in 5% milk/TBS-tween at 4°C overnight. Menin / LC3 blots were then probed with secondary antibody (ab216773, IRDye 800CW) for 2 hours at room temperature, followed by imaging on the ChemiDoc MP system.

4. Supplementary Figures 1-35

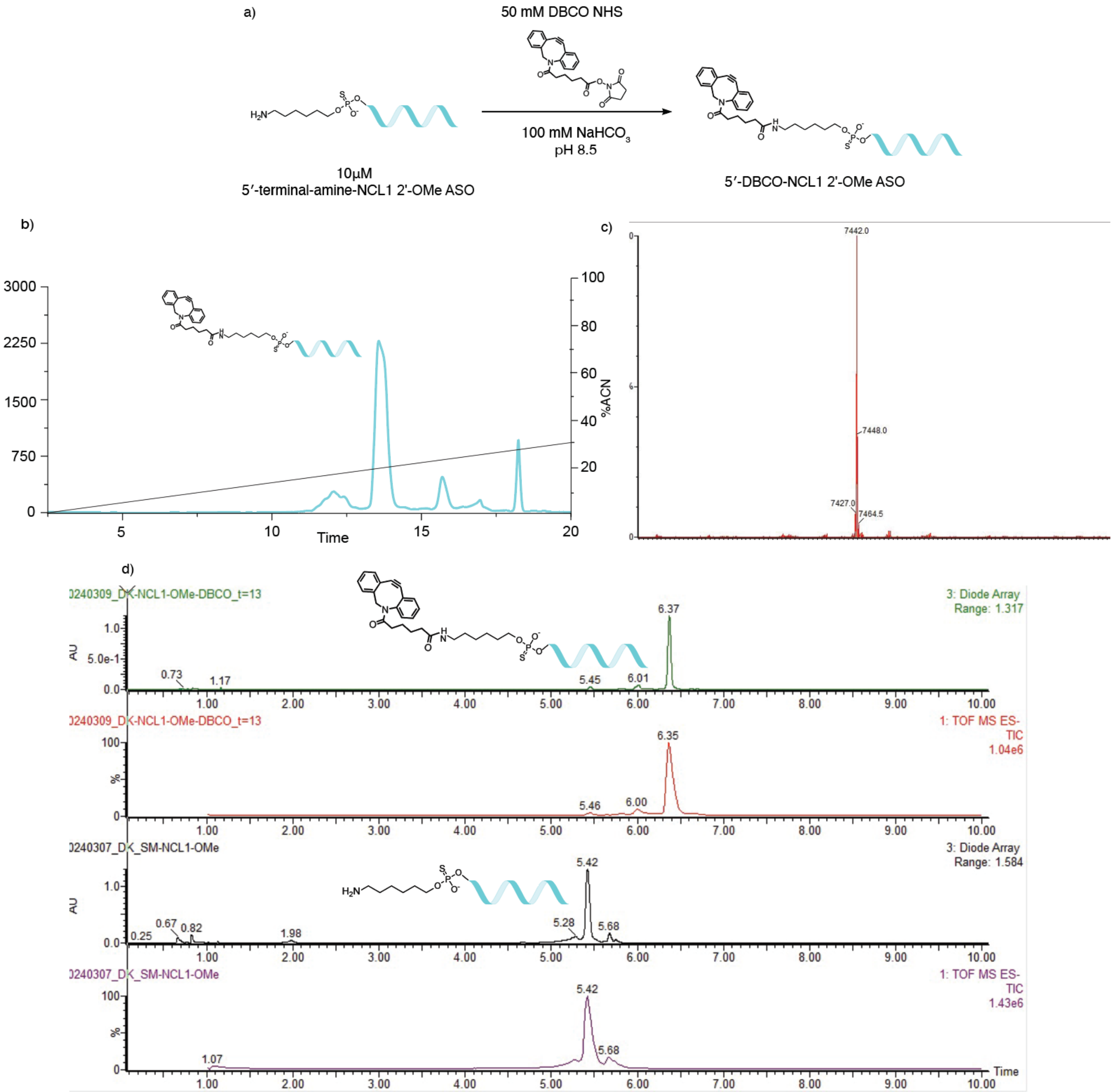

Supplementary Figure 1. Reaction and characterisation for DBCO modification of NCL1 2'-OMe ASO. **a**) Reaction scheme for DBCO functionalisation of NCL1 2'-OMe ASO. **b**) HPLC purification for DBCO-NCL1 2'-OMe ASO functionalisation. **c**) Mass spectrum for HPLC-purified DBCO-NCL1 2'-OMe ASO. **d**) LC-MS characterisation for HPLC-purified DBCO-NCL1 2'-OMe ASO.

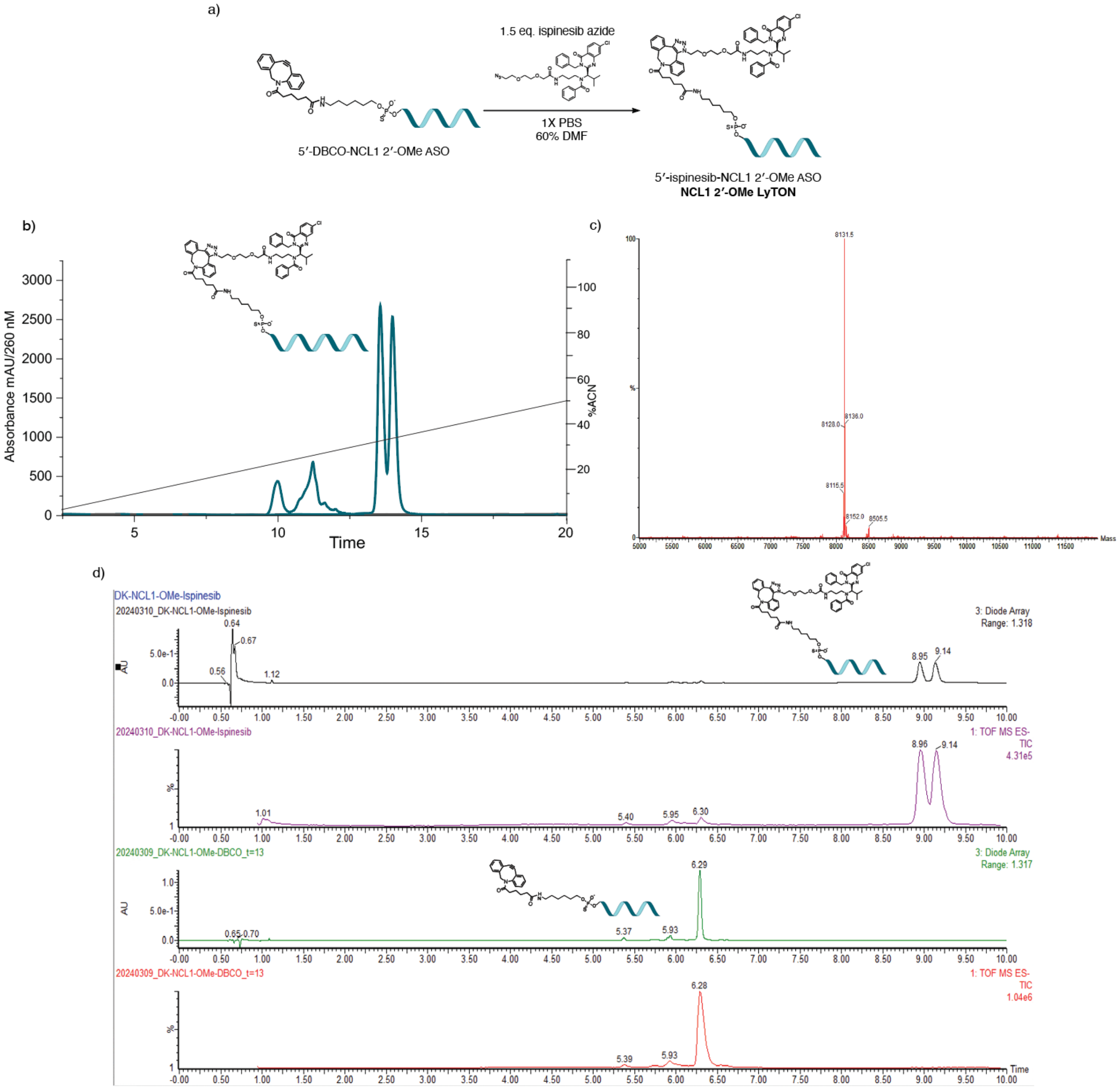

Supplementary Figure 2. Reaction and characterisation for ispinesib modification of NCL1 2'-OMe ASO. **a**) Reaction scheme for ispinesib functionalisation of NCL1 2'-OMe ASO. **b**) HPLC purification for Ispinesib-NCL1 2'-OMe ASO (NCL1 2'-OMe LyTON) functionalisation (two peaks represent regioisomer products from SPAAC). **c**) Mass spectrum for HPLC-purified NCL1 2'-OMe LyTON. **d**) LC-MS characterisation for HPLC-purified NCL1 2'-OMe LyTON.

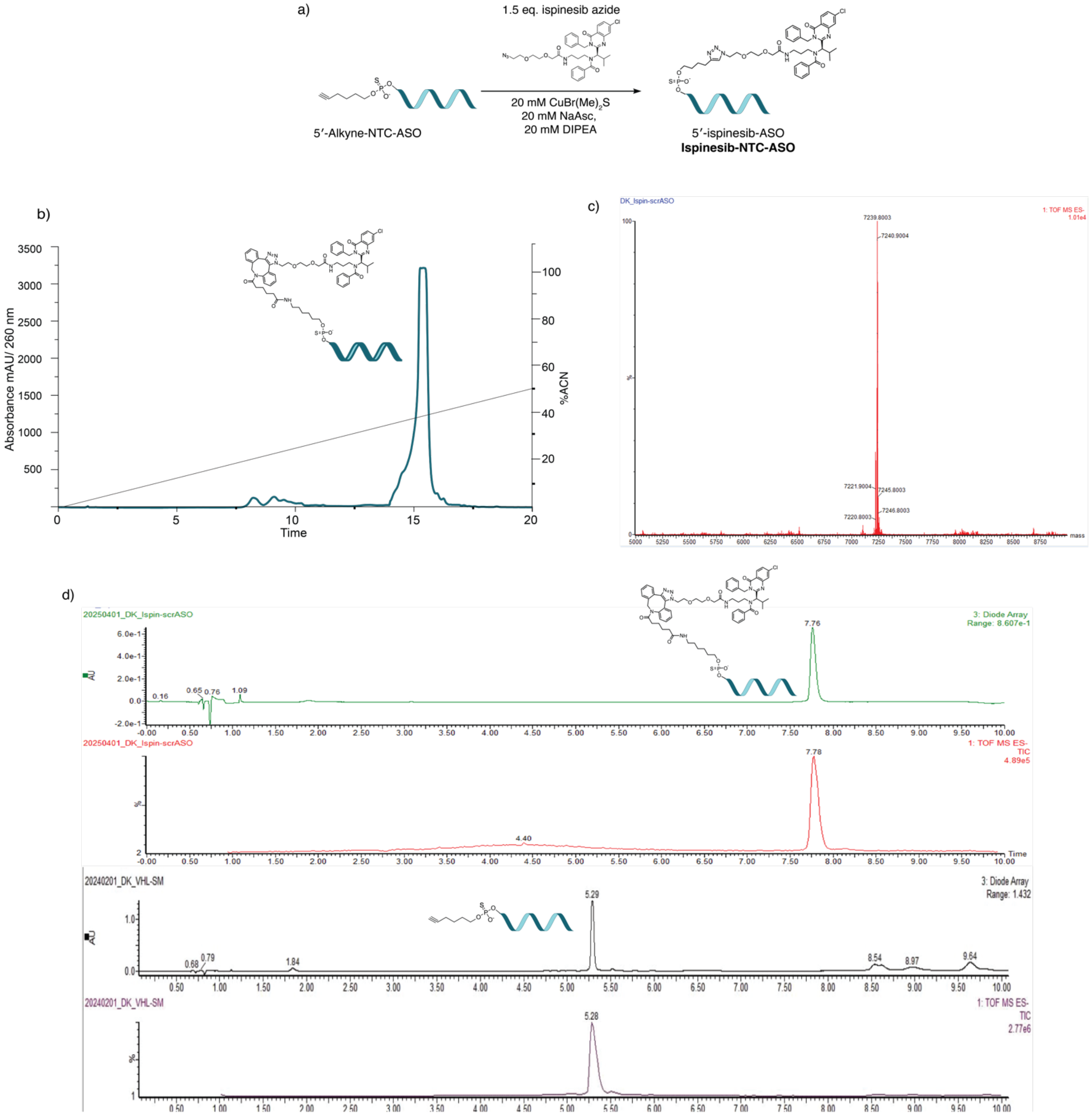

Supplementary Figure 3. Reaction and characterisation for ispinesib modification of NTC-ASO. **a**) Reaction scheme for ispinesib functionalisation of NTC-ASO. **b**) HPLC purification for Ispinesib-NTC-ASO functionalisation. **c**) Mass spectrum for HPLC-purified Ispinesib-NTC-ASO. **d)** LC-MS characterisation for HPLC-purified Ispinesib-NTC-ASO.

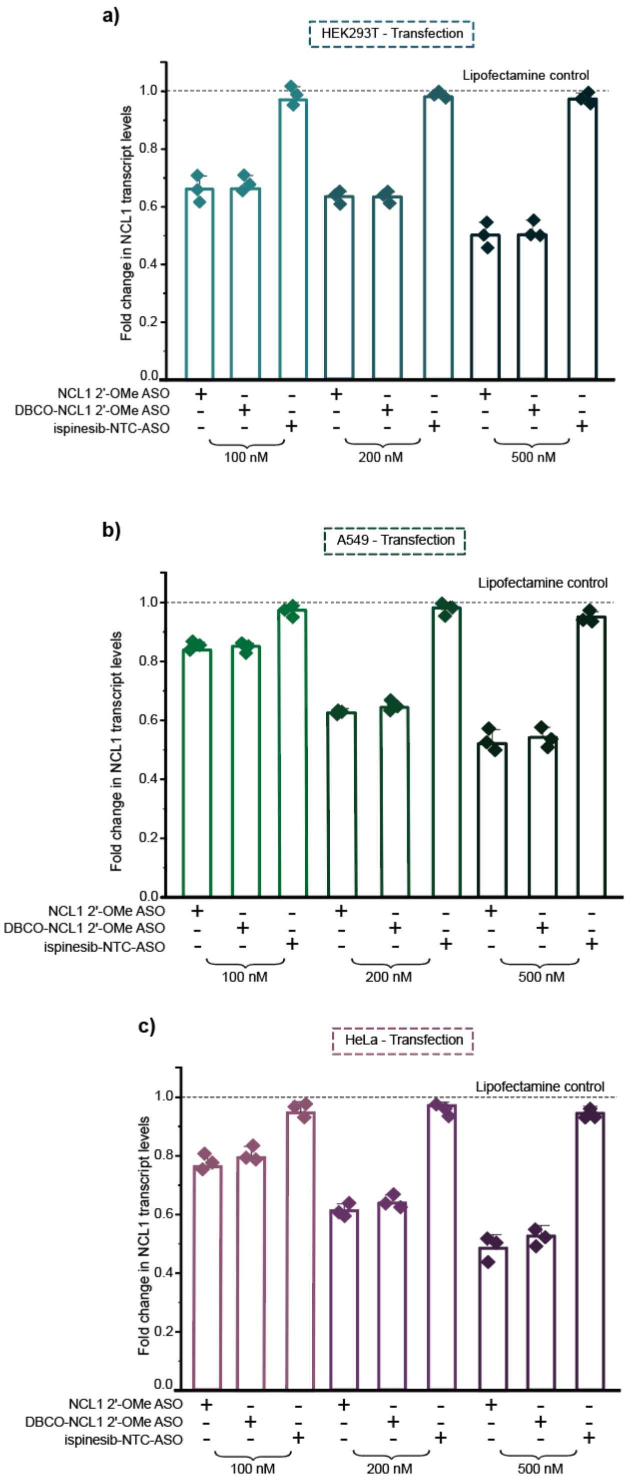

Supplementary Figure 4. RT-qPCR data of NCL1 knockdown upon unmodified NCL1 2'-OMe ASO, DBCO-modified NCL1 2'-OMe ASO and ispinesib-NTC-ASO lipofectamine transfection in a) HEK293Ts, b) A549, c) HeLa for 24 hours at concentrations indicated.

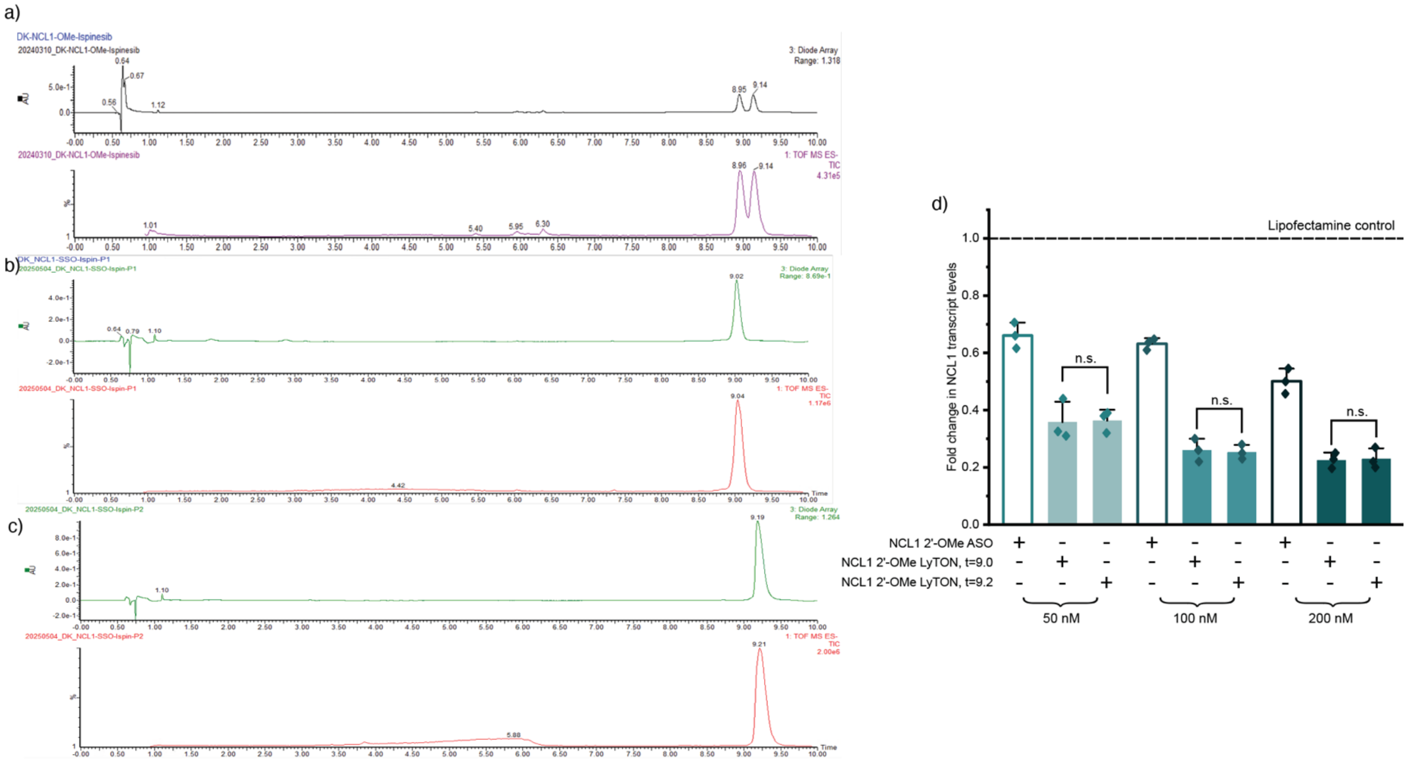

Supplementary Figure 5. LC-MS characterisation and RT-qPCR for regioisomers for ispinesib modification of NCL1 2'-OMe ASO. **a**) LC-MS trace for HPLC-purified NCL1 2'-OMe LyTON regioisomer mixture .**b**) LC-MS trace for HPLC-purified NCL1 2'-OMe LyTON regioisomer, t=9.0. **c**) LC-MS trace for HPLC-purified NCL1 2'-OMe LyTON regioisomer, t=9.2. **d**) RT-qPCR data for NCL1 transcript levels upon lipofectamine transfection with purified regioisomers of NCL1 2'-OMe LyTON at 24 hours at concentrations indicated.

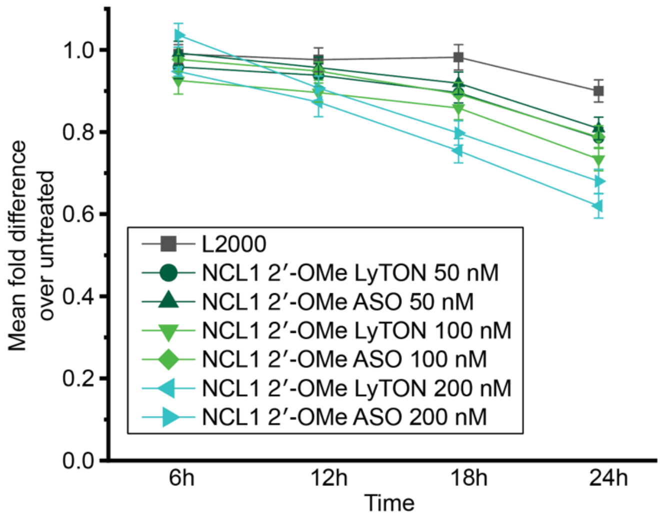

Supplementary Figure 6. Cell viability of the HEK293T upon NCL1 2'-OMe ASO and NCL1 2'-OMe LyTON treatment evaluated by Cell-Titer Glo.

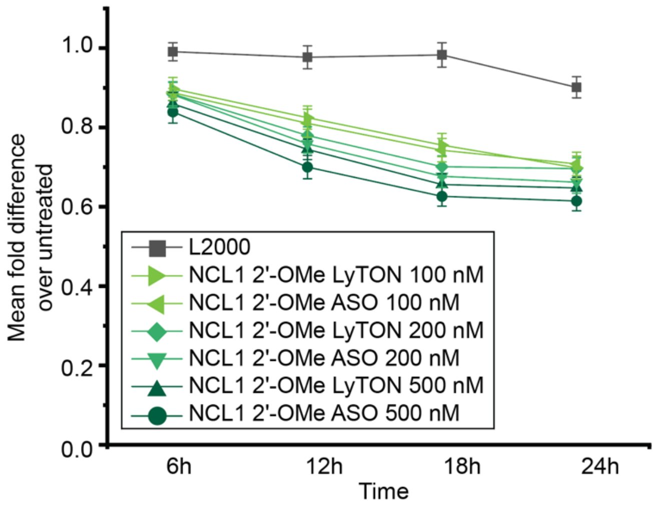

Supplementary Figure 7. Cell viability of the A549 upon NCL1 2'-OMe ASO and NCL1 2'-OMe LyTON treatment evaluated by Cell-Titer Glo.

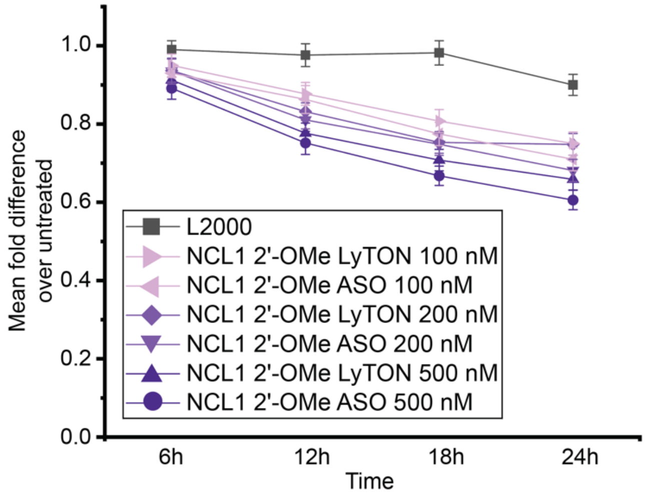

Supplementary Figure 8. Cell viability of the HeLa upon NCL1 2'-OMe ASO and NCL1 2'-OMe LyTON treatment evaluated by Cell-Titer Glo.

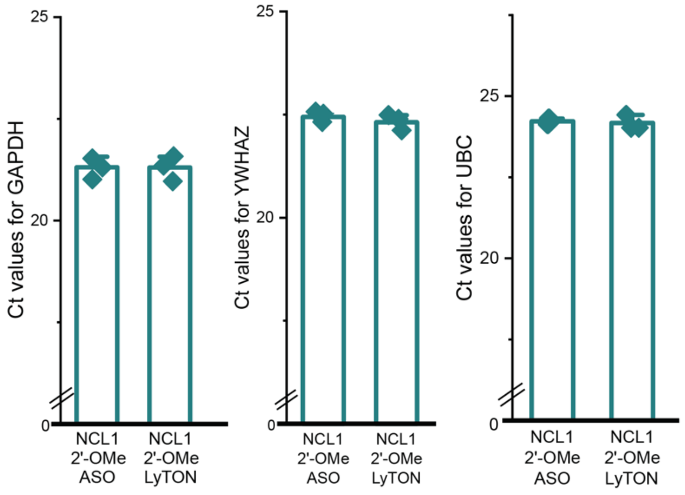

Supplementary Figure 9. Ct values for key housekeeping genes upon NCL1 2'-OMe ASO and NCL1 2'-OMe LyTON treatment at 200 nM in HEK293T.

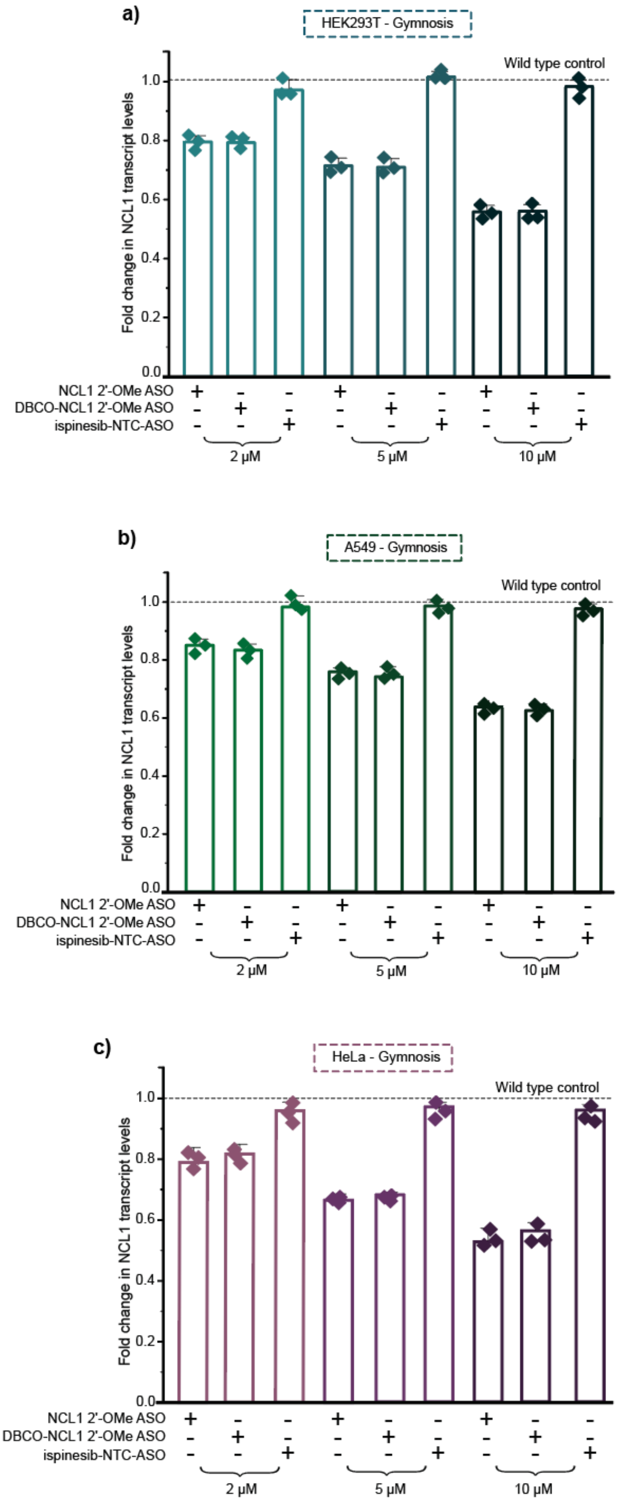

Supplementary Figure 10. RT-qPCR data of NCL1 knockdown upon unmodified NCL1 2'-OMe ASO, DBCO-modified NCL1 2'-OMe ASO and ispinesib-NTC-ASO gymnosis in a) HEK293T, b) A549, c) HeLa for 96 hours at concentrations indicated.

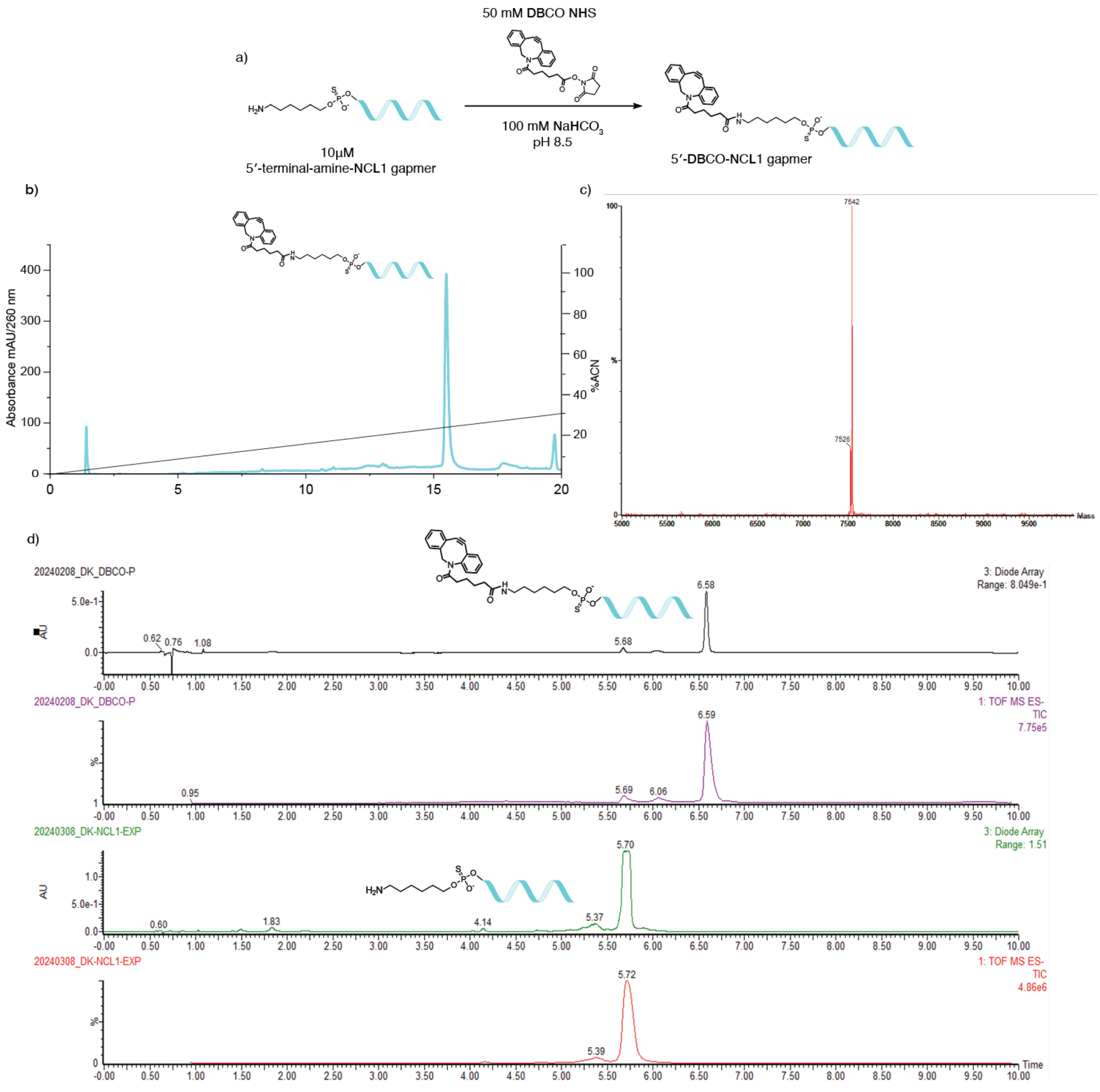

Supplementary Figure 11. Reaction and characterisation for DBCO modification of NCL1 gapmer ASO. **a**) Reaction scheme for DBCO functionalisation of NCL1 gapmer ASO. **b**) HPLC purification for DBCO-NCL1 gapmer ASO functionalisation. **c**) Mass spectrum for HPLC-purified DBCO-NCL1 gapmer ASO. **d**) LC-MS characterisation for HPLC-purified DBCO-NCL1 gapmer ASO.

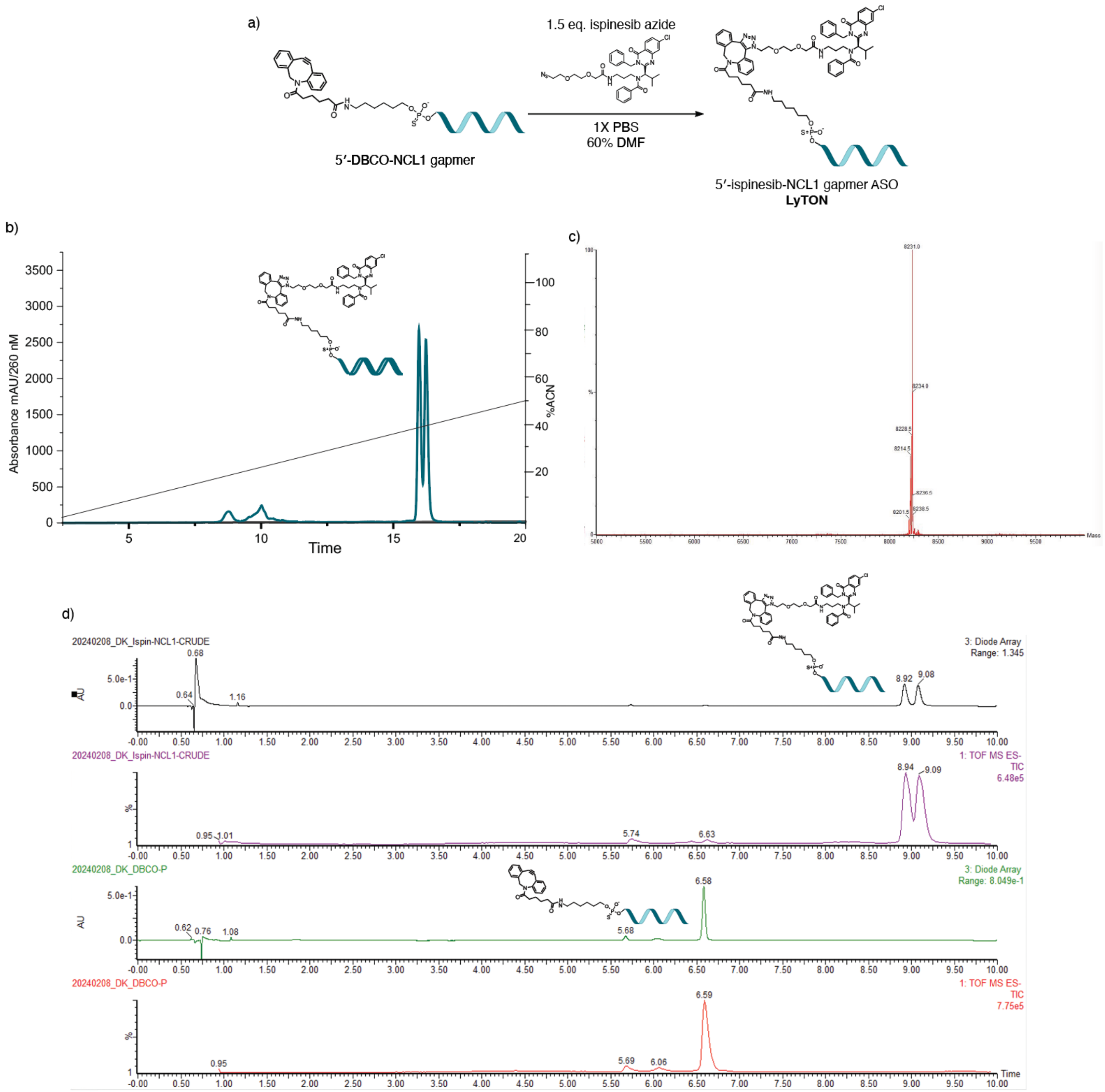

Supplementary Figure 12. Reaction and characterisation for ispinesib modification of NCL1 gapmer ASO. **a**) Reaction scheme for ispinesib functionalisation of NCL1 gapmer ASO. **b**) HPLC purification for Ispinesib-NCL1 gapmer ASO (NCL1 gapmer LyTON) functionalisation (two peaks represent regioisomer products from SPAAC). **c**) Mass spectrum for HPLC-purified NCL1 gapmer LyTON. **d**) LC-MS characterisation for HPLC-purified NCL1 gapmer LyTON.

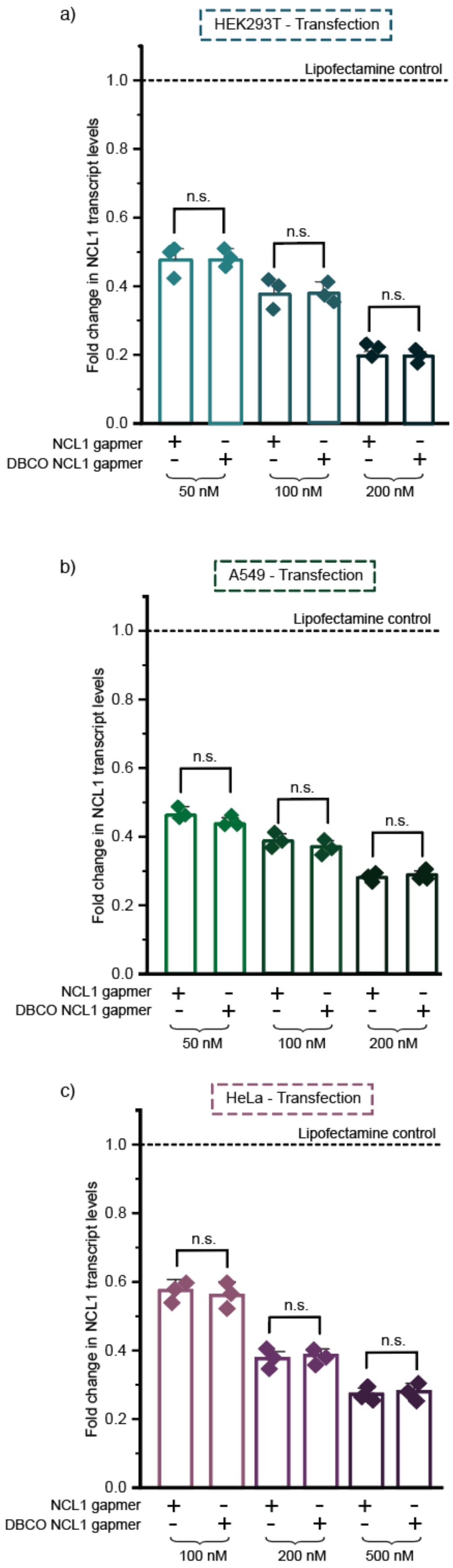

Supplementary Figure 13. RT-qPCR data of NCL1 knockdown upon unmodified and DBCO-modified NCL1 gapmer lipofectamine transfection in a) HEK293T, b) A549, c) HeLa for 24 hours at concentrations indicated.

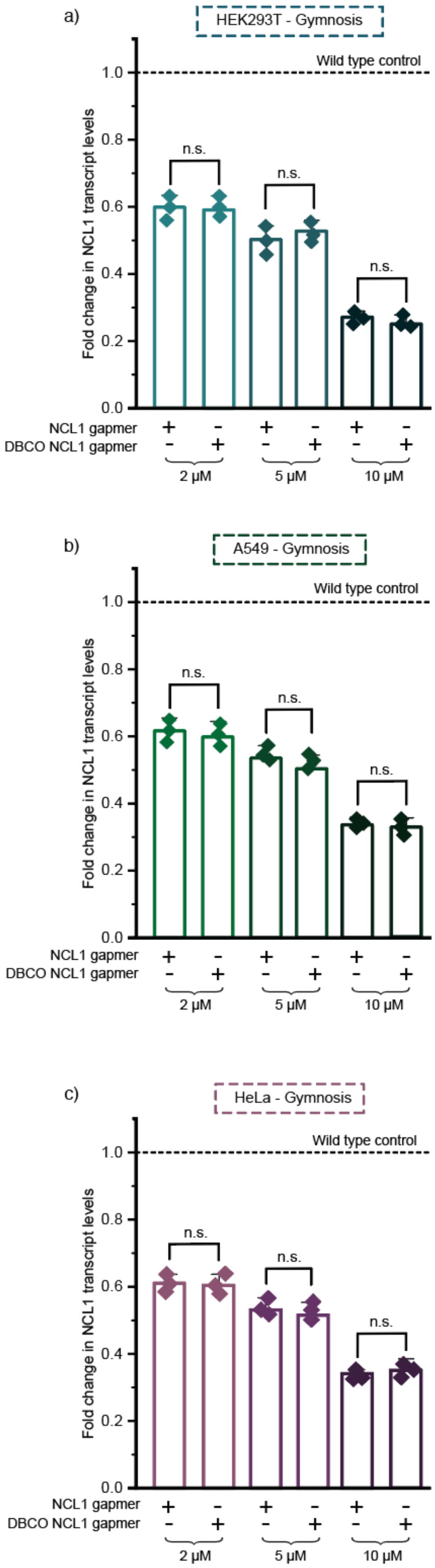

Supplementary Figure 14. RT-qPCR data of NCL1 knockdown upon unmodified and DBCO-modified NCL1 gapmer gymnosis in a) HEK293T, b) A549, c) HeLa for 96 hours at concentrations indicated.

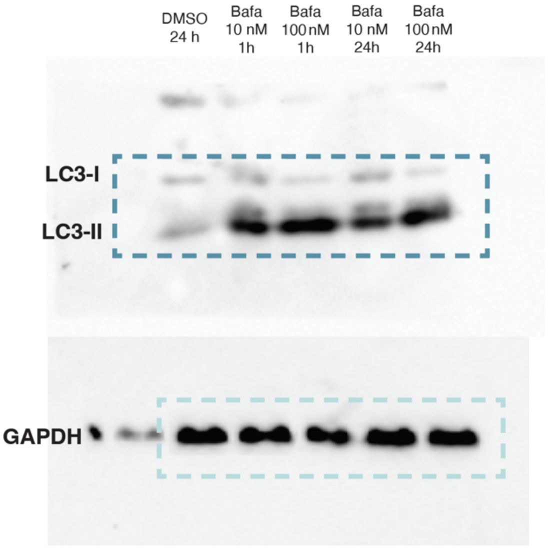

Supplementary Figure 15. Uncropped western blot of LC3-I and LC3-II levels upon treatment with bafilomycin at indicated concentrations and time points in HEK293Ts

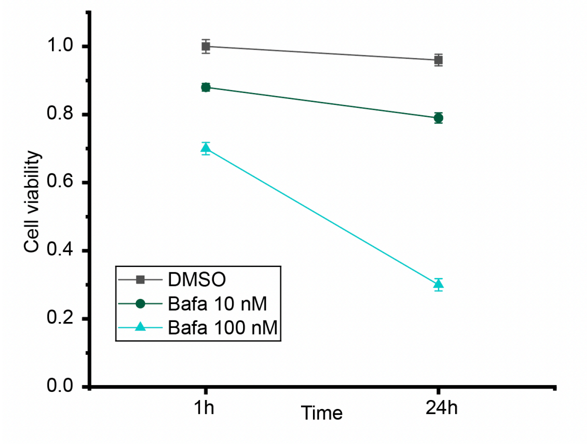

Supplementary Figure 16. Cell viability upon bafilomycin (Bafa) treatment in HEK293Ts assayed by dye exclusion on hemocytometer.

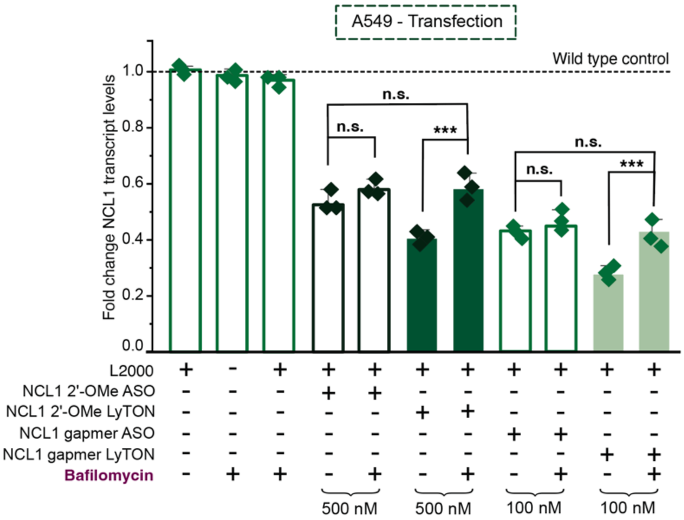

Supplementary Figure 17. RT-qPCR data for NCL1 knockdown upon lipofectamine transfection with NCL1 gapmer ASO, NCL1 gapmer LyTON, NCL1 2'-OMe ASO, and NCL1 2'-OMe LyTON in A549 cells in the presence or absence of 10 nM bafilomycin, at the concentrations indicated.

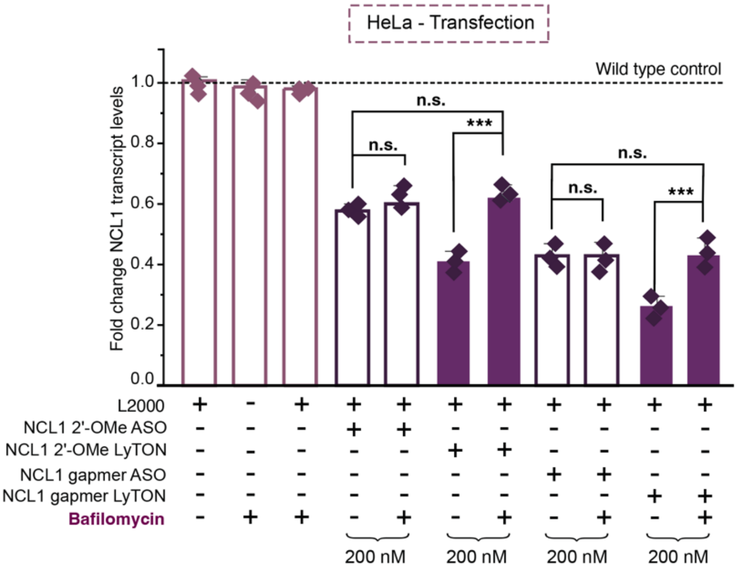

Supplementary Figure 18. RT-qPCR data for NCL1 knockdown upon lipofectamine transfection of NCL1 gapmer ASO, NCL1 gapmer LyTON, NCL1 2'-OMe ASO, and NCL1 2'-OMe LyTON in HeLa cells in the presence or absence of 10 nM bafilomycin, at the concentrations indicated.

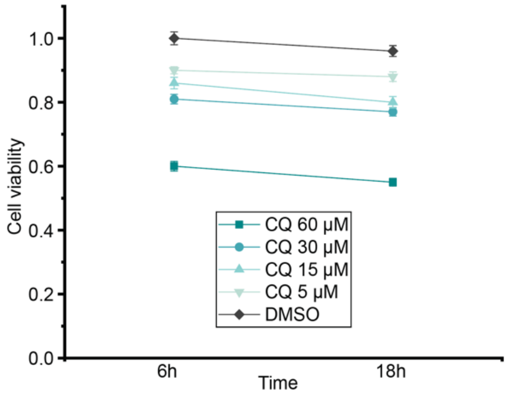

Supplementary Figure 19. Cell viability upon chloroquine (CQ) treatment in HEK293Ts assayed by dye exclusion on hemocytometer.

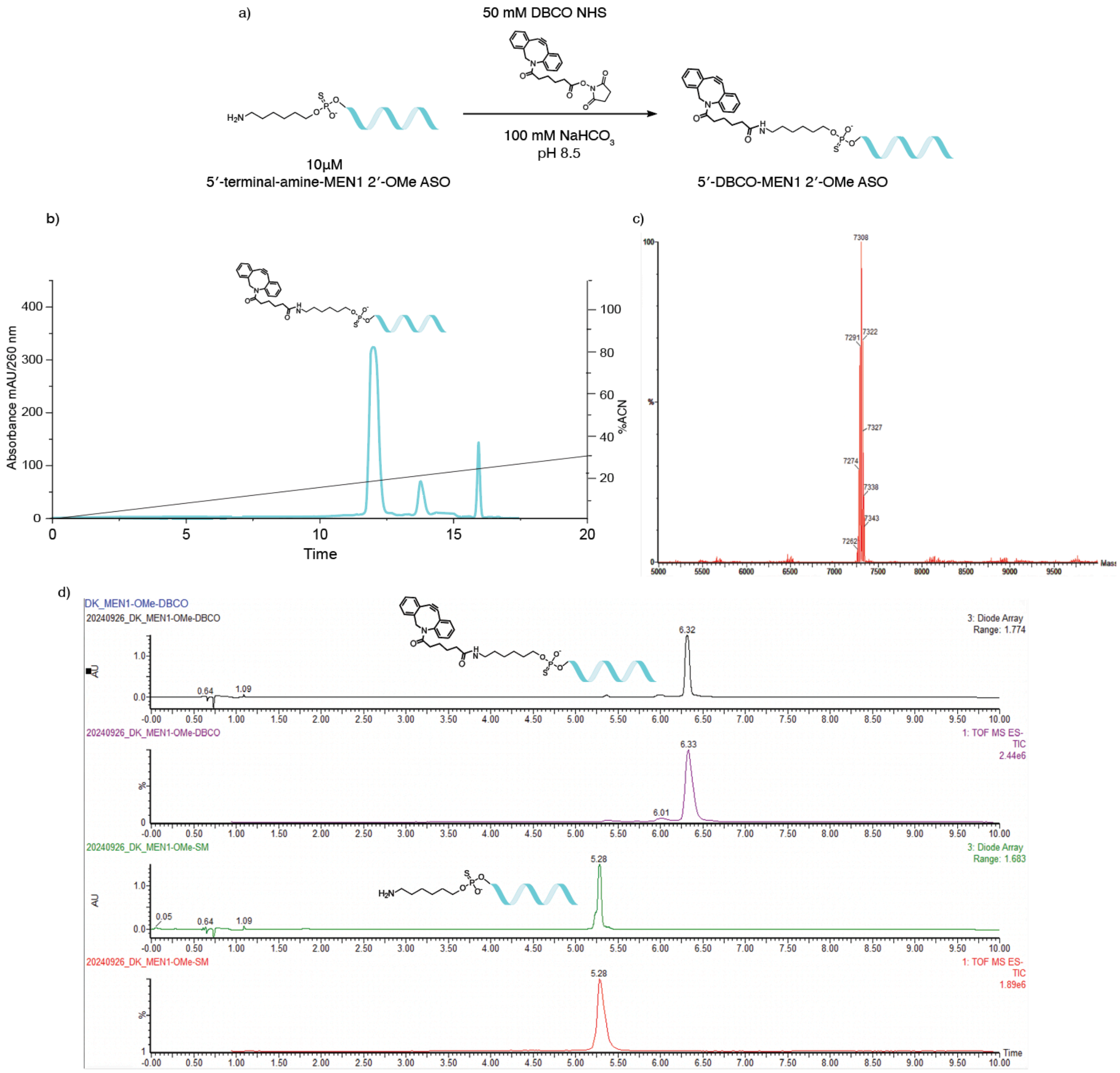

Supplementary Figure 20. Reaction and characterisation for DBCO modification of MEN1 2'-OMe ASO. **a**) Reaction scheme for DBCO functionalisation of MEN1 2'-OMe ASO. **b**) HPLC purification for DBCO-MEN1 2'-OMe ASO functionalisation. **c**) Mass spectrum for HPLC-purified DBCO-MEN1 2'-OMe ASO. **d**) LC-MS characterisation for HPLC-purified DBCO-MEN1 2'-OMe ASO.

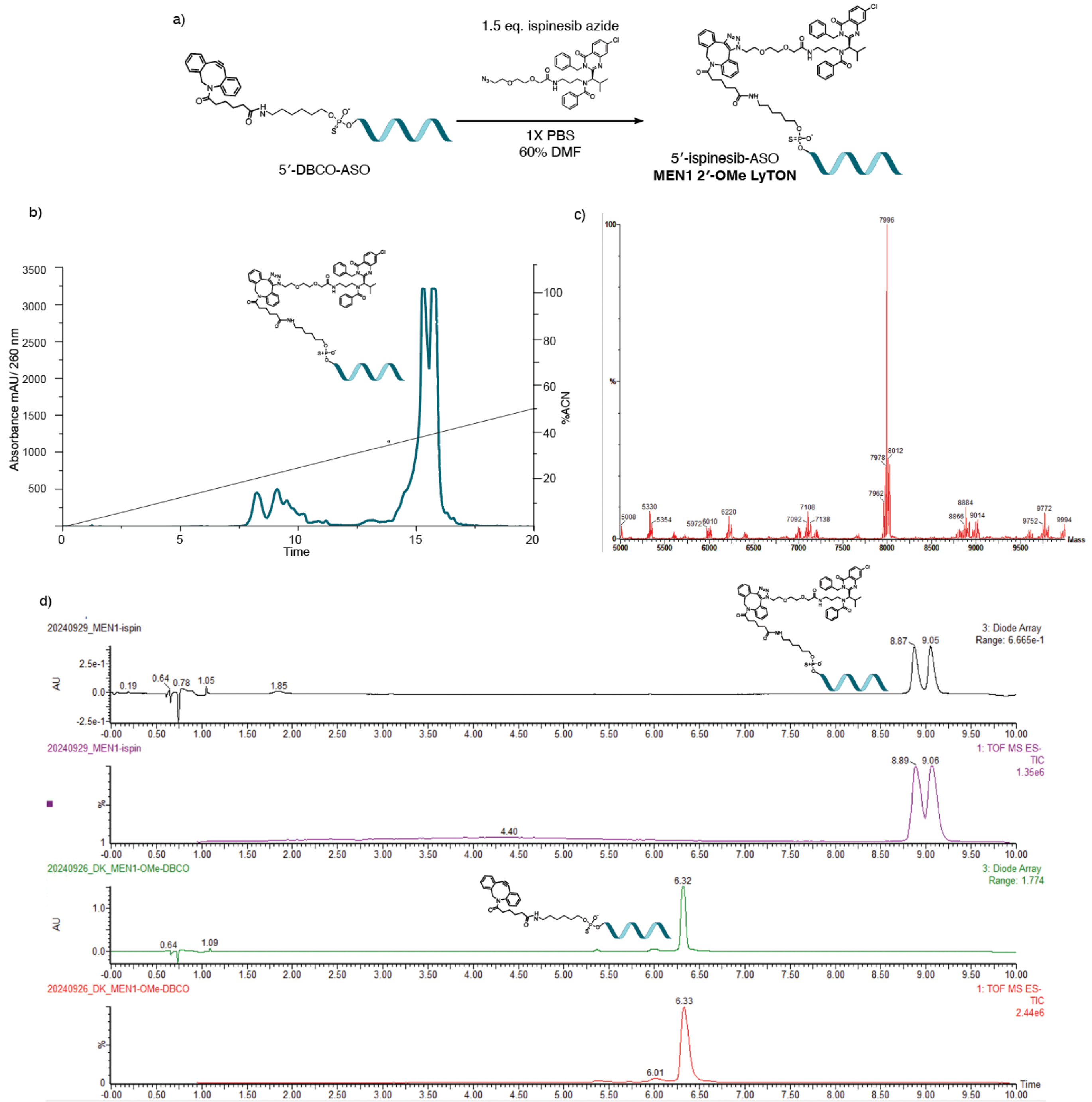

Supplementary Figure 21. Reaction and characterisation for ispinesib modification of MEN1 2'-OMe ASO. **a**) Reaction scheme for ispinesib functionalisation of MEN1 2'-OMe ASO. **b**) HPLC purification for Ispinesib-MEN1 2'-OMe ASO (MEN1 2'-OMe LyTON) functionalisation (two peaks represent regioisomer products from SPAAC). **c**) Mass spectrum for HPLC-purified MEN1 2'-OMe LyTON. **d**) LC-MS characterisation for HPLC-purified MEN1 2'-OMe LyTON.

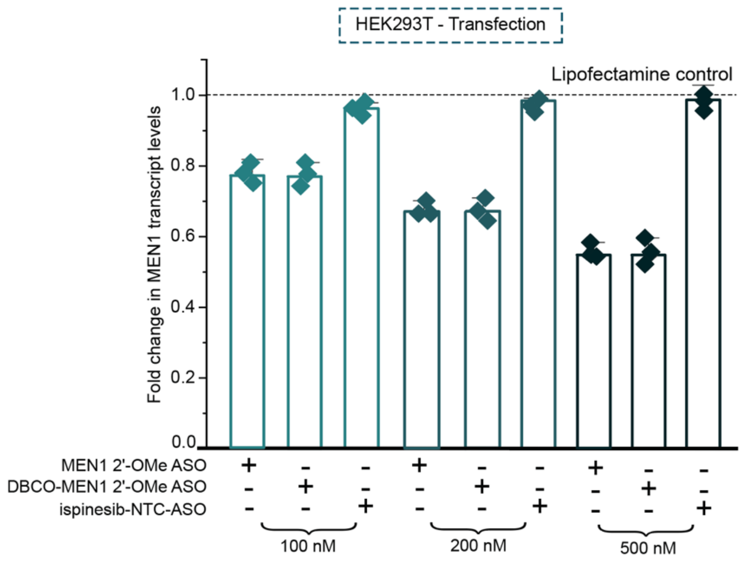

Supplementary Figure 22. RT-qPCR data of MEN1 knockdown upon unmodified MEN1 2'-OMe ASO, DBCO-modified MEN1 2'-OMe ASO and ispinesib-NTC-ASO lipofectamine transfection in HEK293T for 24 hours at concentrations indicated.

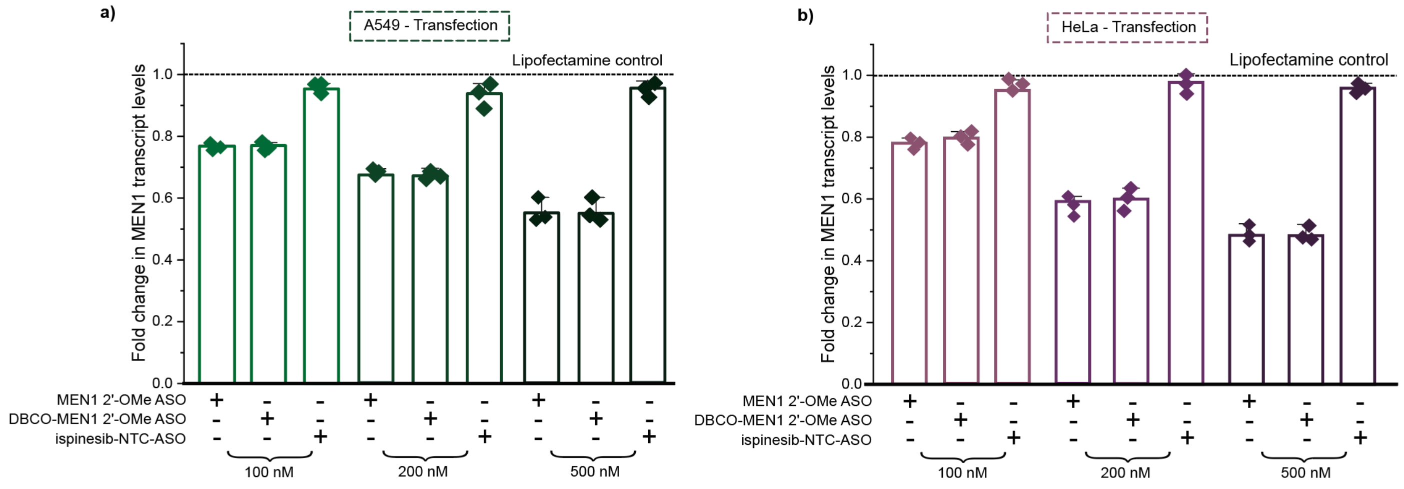

Supplementary Figure 23. RT-qPCR data of MEN1 knockdown upon unmodified MEN1 2'-OMe ASO, DBCO-modified MEN1 2'-OMe ASO and ispinesib-NTC-ASO lipofectamine transfection in a) A549, b) HeLa for 24 hours at concentrations indicated.

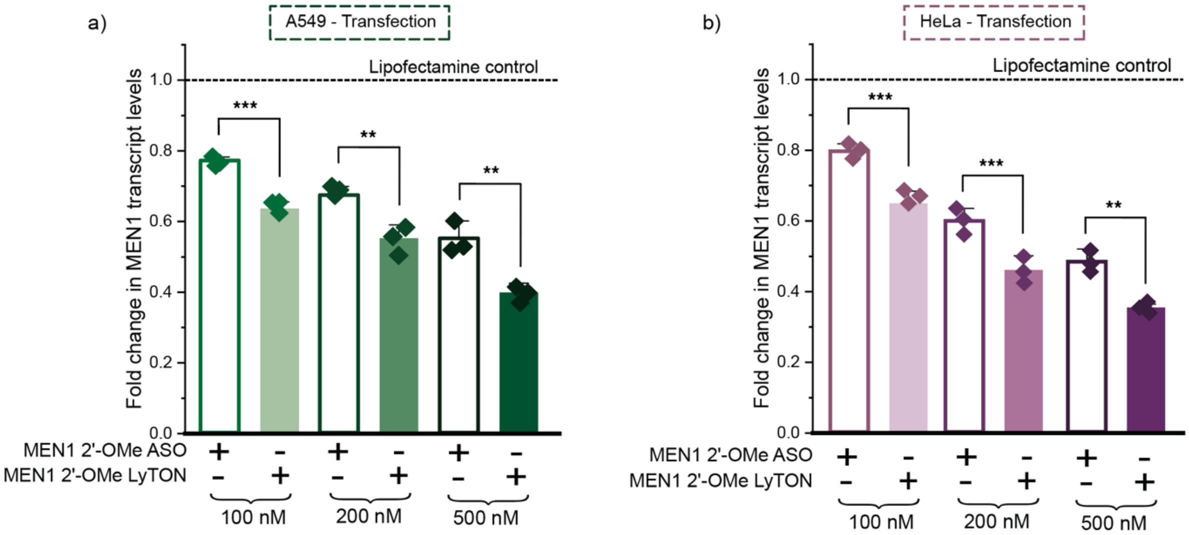

Supplementary Figure 24. RT-qPCR data for MEN1 knockdown upon lipofectamine transfection of unmodified MEN1 2'-OMe ASO and MEN1 2'-OMe LyTON ASO in a) A549, b) HeLa at the concentrations indicated for 24 hours.

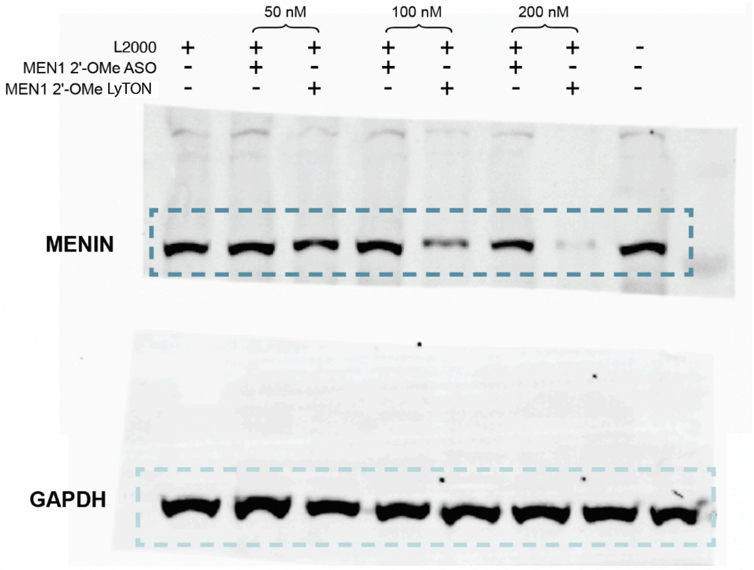

Supplementary Figure 25, Figure 5c. Uncropped western blot of Menin levels upon treatment with MEN1 2'-OMe ASO and MEN1 2'-OMe LyTON upon transfection with lipofectamine, harvested at 48 hours.

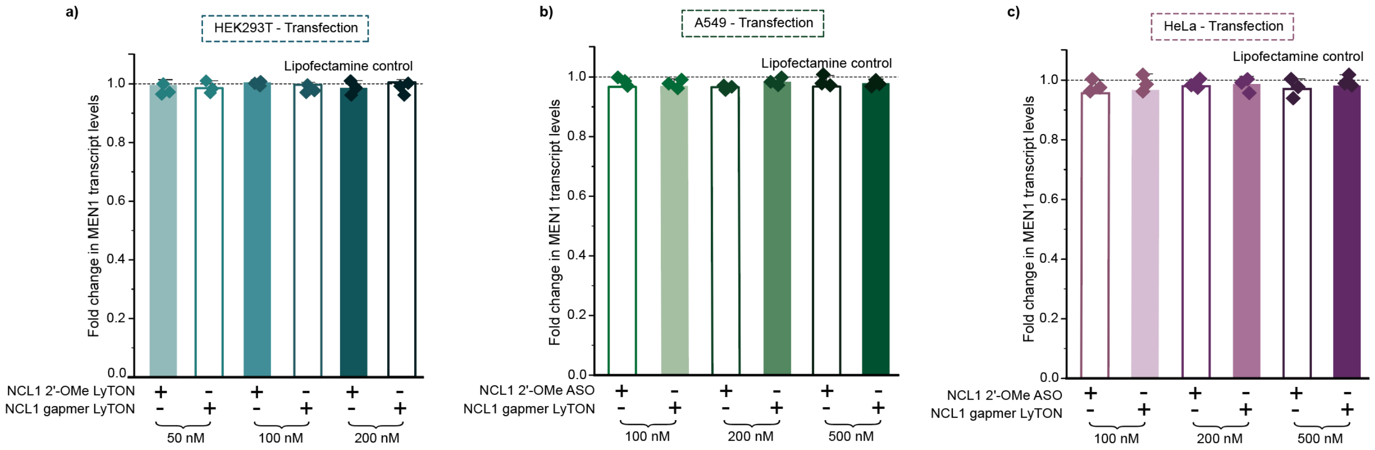

Supplementary Figure 26. RT-qPCR data of MEN1 knockdown upon NCL1 2'-OMe LyTON lipofectamine transfection in a) HEK293T, b) A549, c) HeLa for 24 hours at concentration indicated.

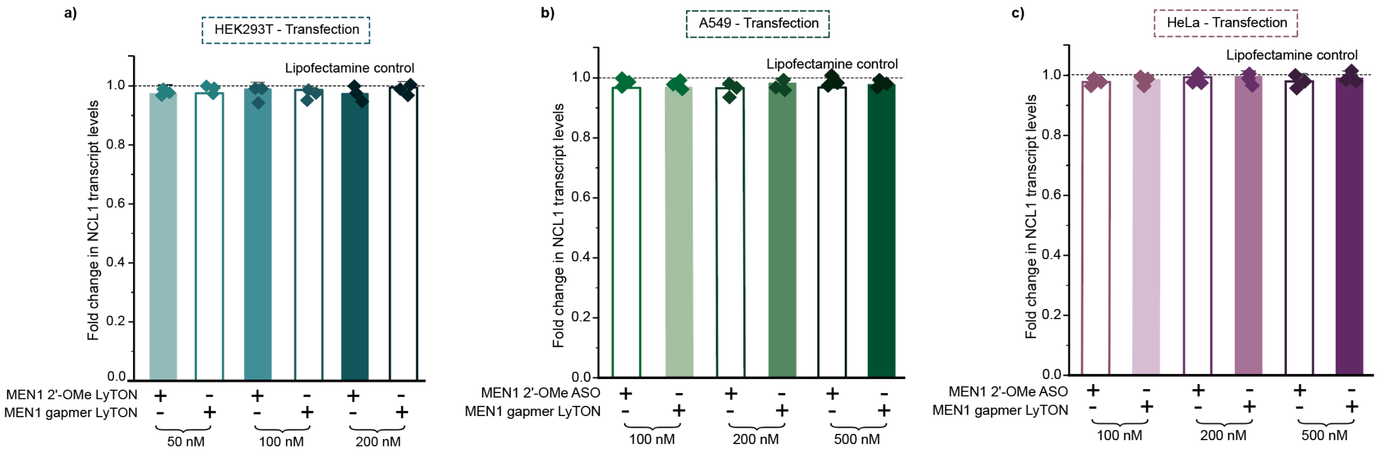

Supplementary Figure 27. RT-qPCR data of NCL1 knockdown upon MEN1 2'-OMe LyTON lipofectamine transfection in a) HEK293T, b) A549, c) HeLa for 24 hours at concentration indicated.

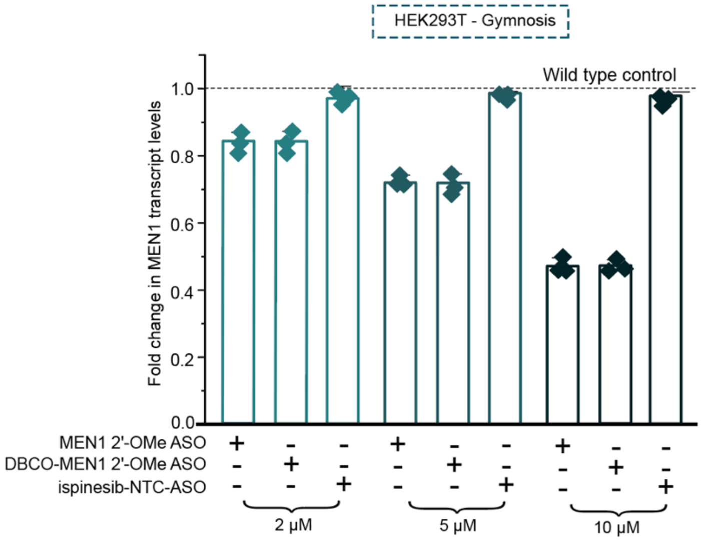

Supplementary Figure 28: RT-qPCR data of MEN1 knockdown upon unmodified MEN1 2'-ASO, DBCO-modified MEN1 2'-OMe ASO and ispinesib-NTC-ASO gymnosis in HEK293T for 96 hours at concentrations indicated.

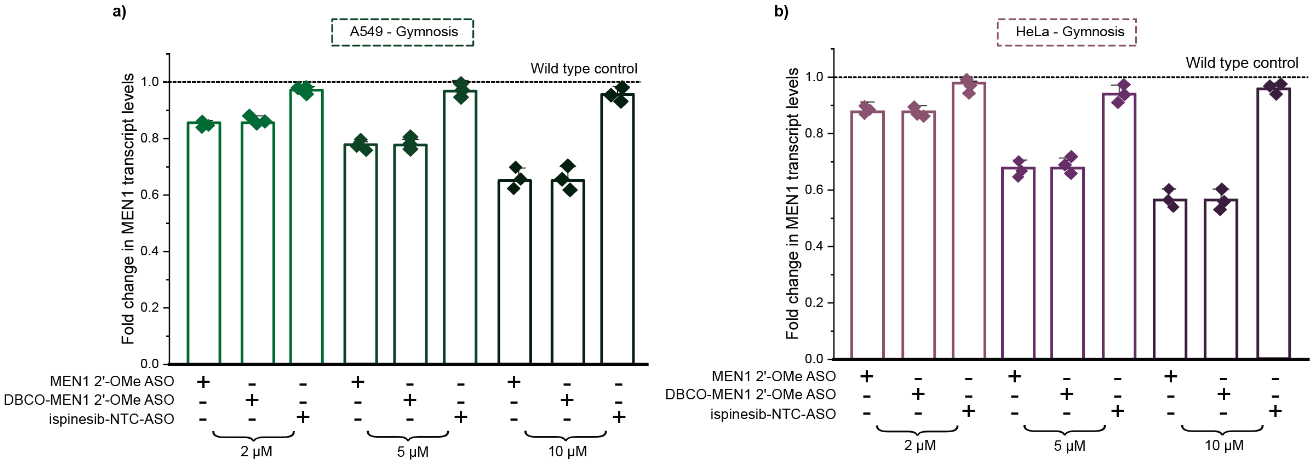

Supplementary Figure 29: RT-qPCR data of MEN1 knockdown upon unmodified MEN1 2'-ASO, DBCO-modified MEN1 2'-OMe ASO and ispinesib-NTC-ASO gymnosis in a) A549, b) HeLa for 96 hours at concentrations indicated.

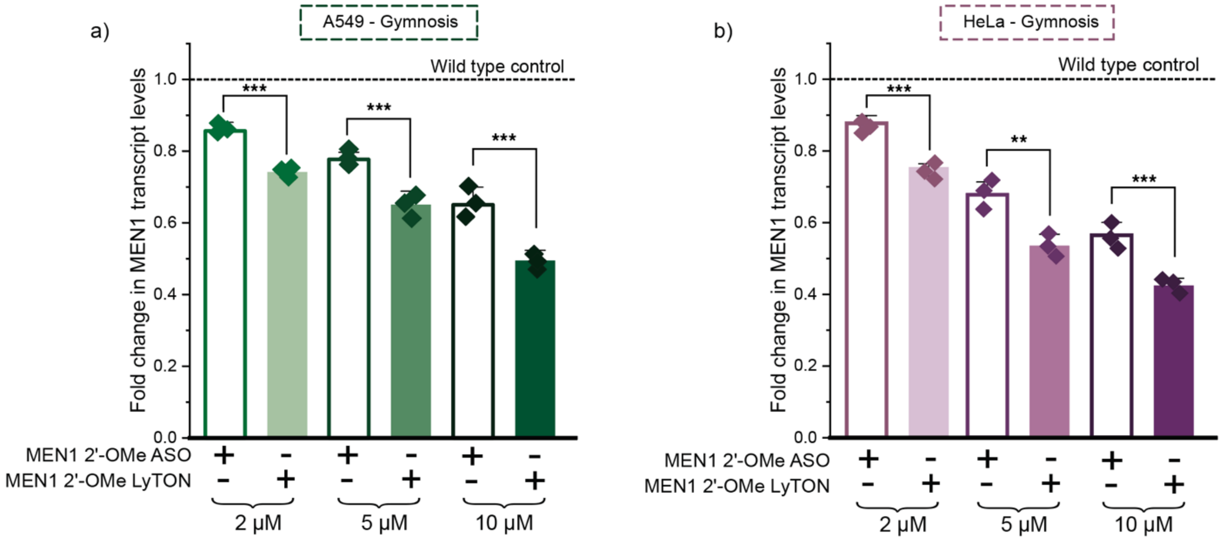

Supplementary Figure 30. RT-qPCR data for MEN1 knockdown upon gymnosis of unmodified MEN1 2'-OMe ASO and MEN1 2'-OMe LyTON ASO in a) A549, b) HeLa at the concentrations indicated for 96 hours.

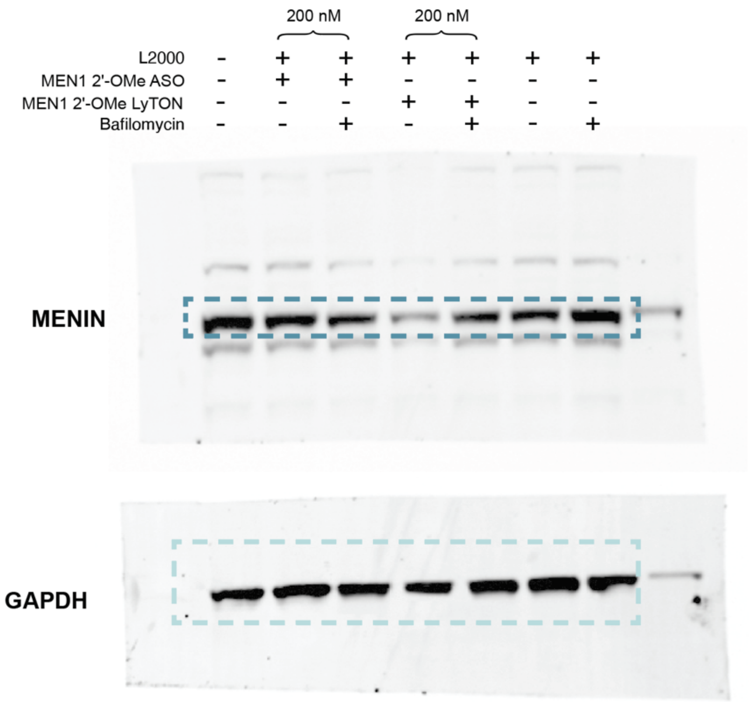

Supplementary Figure 31, Figure 5f. Uncropped western blot of Menin levels upon treatment with MEN1 2'-OMe ASO and MEN1 2'-OMe LyTON – in presence and absence of 10 nM bafilomycin, upon transfection with lipofectamine, harvested at 48 hours.

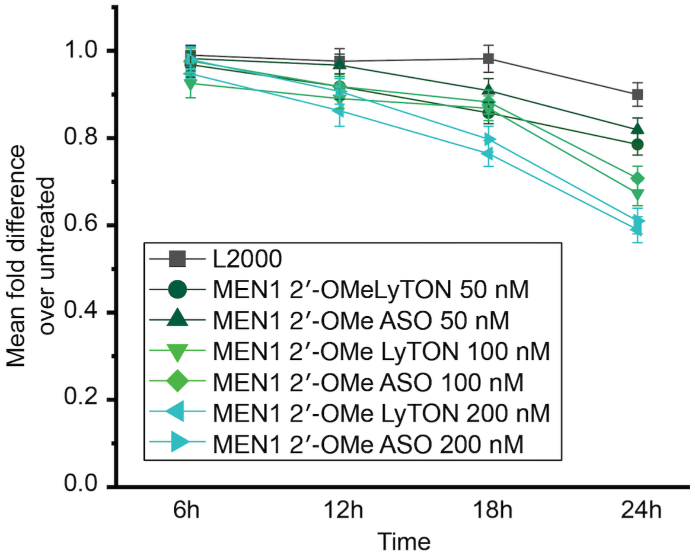

Supplementary Figure 32. Viability of HEK293T cells upon MEN1 2'-OMe ASO and MEN1 2'-OMe LyTON treatment evaluated by Cell-Titer Glo.

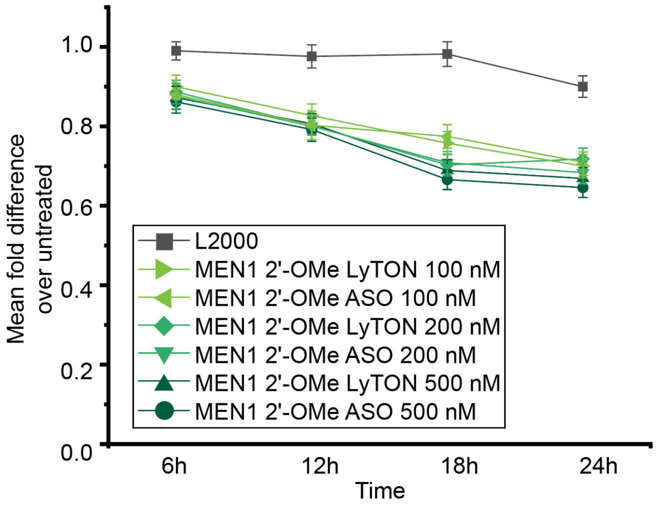

Supplementary Figure 33. Viability of A549 cells upon MEN1 2'-OMe ASO and MEN1 2'-OMe LyTON treatment evaluated by Cell-Titer Glo.

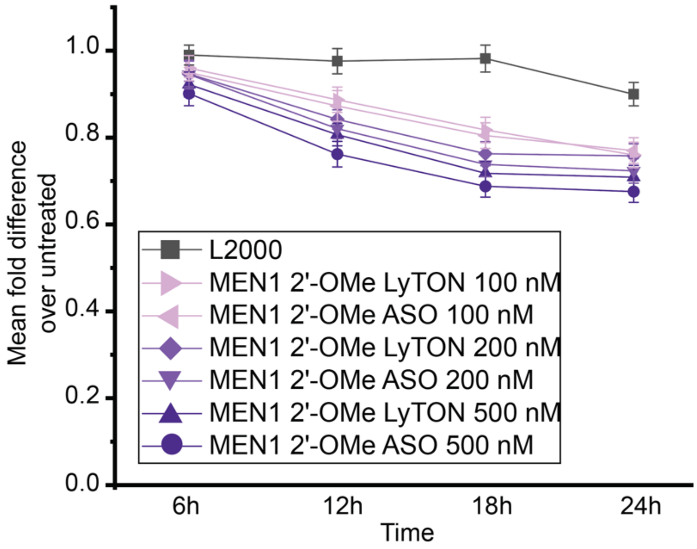

Supplementary Figure 34. Viability of HeLa cells upon MEN1 2'-OMe ASO and MEN1 2'-OMe LyTON treatment evaluated by Cell-Titer Glo.

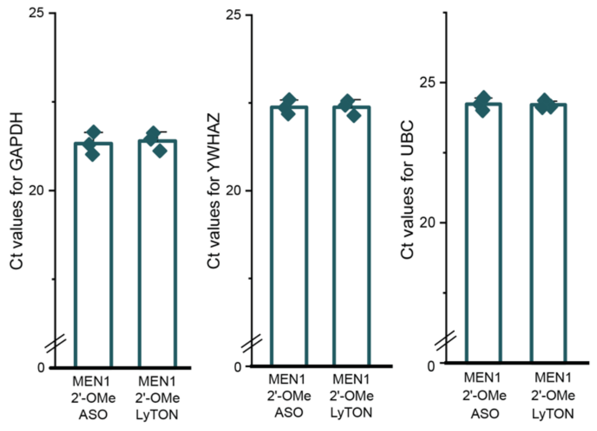

Supplementary Figure 35. Ct values for key housekeeping genes upon MEN1 2'-OMe ASO and MEN1 2'-OMe LyTON treatment at 200 nM.

References

(1) Wilkinson, A. C.; Ballabio, E.; Geng, H.; North, P.; Tapia, M.; Kerry, J.; Biswas, D.; Roeder, R. G.; Allis, C. D.; Melnick, A.; et al. RUNX1 is a key target in t(4;11) leukemias that contributes to gene activation through an AF4-MLL complex interaction. *Cell Rep* **2013**, *3* (1), 116-127. DOI: 10.1016/j.celrep.2012.12.016 From NLM.
